# Biologics in IgE-mediated food allergy: A systematic review and meta-analysis of interventional studies^☆^

**DOI:** 10.1016/j.waojou.2025.101069

**Published:** 2025-05-27

**Authors:** Ulugbek B. Nurmatov, Lucia Lo Scalzo, Francesca Galletta, Marianna Krasnenkova, Stefania Arasi, Ignacio J. Ansotegui, Nara Tagiyeva-Milne, Alessandro Fiocchi

**Affiliations:** a Division of Population Medicine, School of Medicine, Cardiff University, Wales, UK; b University of Palermo, Italy; c University of Messina, Italy; d Tashkent Medical Academy, Uzbekistan; e Pediatric Allergology Unit, Bambino Gesù Hospital (IRCCS), Rome, Italy; f Department of Allergy and Immunology, Hospital Quironsalud Bizkaia, Bilbao, Spain; g Liverpool School of Tropical Medicine, England, UK

**Keywords:** Biologics, IgE-mediated food allergy, Systematic review, Meta-analysis, Interventional studies

## Abstract

**Background and aims:**

IgE-mediated food allergy (FA) is a major healthcare problem, affecting millions of children and adults worldwide. FA management usually involves elimination diets; however, there is increasing interest in alternative strategies that enable individualized optimal approaches. Yet, there is little consensus on the optimal strategies for managing FA. This review aimed to evaluate the effectiveness and safety of biologics, including omalizumab (OMA), as monotherapy or in combination with oral immunotherapy (OIT), for FA management.

**Methods:**

A systematic review (SR) and meta-analysis (MA) was conducted, searching 10 international electronic databases (from their start to May 2024) for randomized controlled trials (RCTs) assessing biologics in FA patients. The outcomes were desensitization, increased tolerated dose of food allergens, sustained unresponsiveness, adverse events/reactions (ARs/AEs), quality of life (QoL) measures, immunological biomarkers, and cost-effectiveness. Data were pooled using random-effects model. The study quality was assessed by the Cochrane Risk of Bias.

**Results:**

We included 11 RCTs, 2 secondary reports from earlier RCTs and 2 US National Clinical Trials with 1010 participants in total. Nine RCTs were at low, 3 at moderate, and 1 at high risk of bias. Meta-analyses demonstrated that OMA significantly improved desensitization rates and increased food tolerance thresholds compared to placebo (risk ratio (RR) 2.035, 95% CI: 1.29 to 3.22 and RR 4.90, 95% CI 2.14 to 11.20, respectively.) OMA reduced the risk of food allergic reactions (RR 0.55, 95% CI 0.36 to 0.85) without significantly increasing skin (RR = 1.09, 95% CI 0.45 to 2.65) or other adverse or severe reactions. Immunologic outcomes showed decreased hypersensitivity, a lowered allergic and inflammatory response. QoL measures improved for patients and parents with multifood oral immunotherapy. However, no studies investigated the cost-effectiveness of biologics in FA management.

**Conclusions:**

Based on the existing literature and our SR and MA, OMA can be recommended for use in carefully selected patients with IgE-mediated food allergies as monotherapy. However, patient-specific factors need to be addressed to reduce the risk of food-induced allergic reactions. OMA in combination with oral immunotherapy is recommended for cow's milk allergy. For the other foods, it will be recommended based on the results of ongoing, large RCTs in the field of biologics for food allergy. In order to recommend a wider indication for use, more research is needed to evaluate optimal treatment durations, long-term outcomes, and cost-effectiveness.

## Background and rationale

IgE-mediated food allergy (FA) is a major healthcare problem, affecting millions of children and adults worldwide. The estimated prevalence of FA, is 1–11% of the population worldwide, with a higher prevalence in children.^1^, ^2^, ^3^ The incidence of FA has been increasing in recent decades, particularly in industrialised nations, leading to substantial economic burdens and reduced quality of life for affected individuals and their families.^4^, ^5^, ^6^, ^7^, ^8^

Traditionally, the cornerstone of FA management has been elimination diet of the causative allergens and rescue medications such as antihistamines and epinephrine to treat reactions to accidental exposure followed by anaphylaxis.^9^ However, these approaches are often challenging to maintain, anxiety-provoking, and do not address the underlying immunological mechanisms of the disease. Consequently, there is a growing interest in developing more proactive therapeutic strategies to induce desensitization or tolerance in food-allergic individuals.^10^

Recent advances in our understanding of the immunopathogenesis of FA have highlighted the central role of IgE and its high-affinity receptor FcεRI, as well as mast cells and basophils, cytokines and chemokines, eosinophils, lipid mediators like leukotrienes and prostaglandins, and Th2 and B cells in mediating allergic responses.^11^ These insights have led to the exploration of biologics – particularly monoclonal antibodies targeting key components of the allergic cascade – as potential therapeutic agents to modulate immune responses, reduce allergic inflammation, and improve overall outcomes for patients with FA.^12^, ^13^, ^14^, ^15^ Biologics, such as omalizumab (OMA), dupilumab, and mepolizumab, have shown promise in FA treatment. For example, OMA has been shown to bind to free IgE, preventing it from activating mast cells and basophils. Dupilumab inhibits IL-4 and IL-13 signalling, reducing inflammation and IgE production. Mepolizumab, reslizumab, and benralizumab modulate IL-5 pathways, reducing eosinophil levels. Tezepelumab targets thymic stromal lymphopoietin (TSLP), disrupting upstream activation of the allergic response. By targeting these specific components, monoclonal antibodies offer promising therapeutic options for managing FA. Concurrently, oral immunotherapy (OIT) has shown promise in inducing desensitization and long-term tolerance in some patients.^16^^,^^17^

The use of biologics, either as monotherapy or in combination with OIT, offers a cutting-edge approach to FA management. However, despite the growing body of research, the optimal use of biologics in FA treatment, including timing, duration, combination strategies, and cost-effectiveness remains to be fully elucidated.^18^, ^19^, ^20^, ^21^, ^22^, ^23^, ^24^, ^25^, ^26^, ^27^, ^28^, ^29^, ^30^, ^31^

To this end, some meta-analyses have already been performed in relation to specific aspects, such as the effectiveness of OMA in desensitization to cow's milk^18^ or in their entirety. In these meta-analyses, together with randomized studies, quasi-experimental trials^27^^,^^29^ and/or observational studies^28^ have been included, limiting the robustness of conclusions.

## Methods

This systematic review and meta-analysis focus exclusively on RCTs to provide higher-quality evidence. We aim to comprehensively assess the effectiveness and cost-effectiveness of biologics as monotherapy or in conjunction with OIT, in children and adults with IgE-mediated FA. We evaluated the effectiveness and side effect profiles of these interventions based on The Core Outcome Measures for Food Allergy (COMFA).^32^ Additionally, we examined the impact of these therapies on quality-of-life indices and analyse available pharmaco-economic data to provide a holistic view of their potential benefits and limitations. This review also applies the Grading of Recommendations Assessment, Development and Evaluation (GRADE) approach to assess the certainty of evidence concerning the use of biologics in FA. By synthesizing the latest evidence, this review seeks to inform clinical decision-making, guide research priorities, and ultimately improve patient care in the rapidly evolving field of FA management.

The review was conducted and reported according to the Preferred Reporting Items for Systematic Reviews and Meta-analyses (PRISMA) 2020 guidelines.^33^ The PRISMA checklist is provided in Supplementary Materials (Table E1).

### Search strategy

We systematically searched 10 international electronic databases from the beginning of their existence to May 2024: AMED (1985–2024), CAB (1910–2024), CINAHL (1937–2024), Cochrane Library (1992–2024), EMBASE (1980–2024), Global Health (1987–2024), ISI Web of Science (1970–2024), MEDLINE (1966–2024), Scopus (2004–2024), and TRIP (2003–2024).

The search strategy was initially developed for MEDLINE and EMBASE using controlled vocabulary terms (MeSH and EMTREE) combined with free-text terms using Boolean operators, then adapted for other databases. The full search strategy is provided in the Supplementary Materials (Search strategies 1 and 2). No language or geographic restrictions were applied.

To identify unpublished and ongoing studies, we searched: Current Controlled Trials (www.controlled-trials.com), ClinicalTrials.gov, Australian New Zealand Clinical Trials Registry (www.anzctr.org.au), and WHO International Clinical Trials Registry Platform (ICTRP) (Supplementary materials, table E2). Reference lists of included studies were hand-searched for additional eligible studies and 4 international experts in food allergy research were contacted for potentially relevant unpublished work.

### Eligibility criteria

#### Inclusion criteria

Population: Children (≤18 years) and adults (>18 years) with IgE-mediated food allergy confirmed by oral food challenge.

Intervention: Biological therapy (monotherapy or combined with oral immunotherapy).

Comparator: Placebo, no intervention, or routine management without active treatment.

Primary Outcomes: 1) desensitization (ability to consume, as a result of the intervention, a prespecified amount of food containing the trigger allergen without allergic symptoms or increased food allergen tolerance threshold); 2) sustained unresponsiveness or persistent desensitization (ability to safely consume without restriction a food containing the trigger allergen for at least 26 weeks after discontinuation of treatment); and 3) biologics-related adverse reactions (ARs), including severe adverse events (AEs), as defined by European Medicines Agency (EMA) (https://www.ema.europa.eu/en/glossary-terms/adverse-drug-reaction, accessed November 24, 2024).

Secondary Outcomes: 1) immunological outcomes, namely skin prick testing (SPT) reactivity, serum specific IgE and IgG4 concentrations, and total IgE levels; 2) quality of life (QoL) measures, defined as evaluations of the patient's perception of their position in life in the context of the culture and value systems in which they live, and in relation to their goals, expectations, standards and concerns. This multi-domain construct encompasses at least a physical, a mental and a social health dimension, and is evaluated using validated instruments as the Food Allergy Quality of Life (FAQOL) questionnaire; and 3) cost-effectiveness measures defined as the financial impact of medication costs, food-related expenses and non-health-related costs associated with FA.

Study Design: Randomized controlled trials (RCTs), including cluster RCTs.

#### Exclusion criteria

Observational studies (eg, cohort, case-control, cross-sectional designs), case series and case reports, conference abstracts, non-research letters and editorials were excluded from the review.

### Study selection

**Fig. 1:**
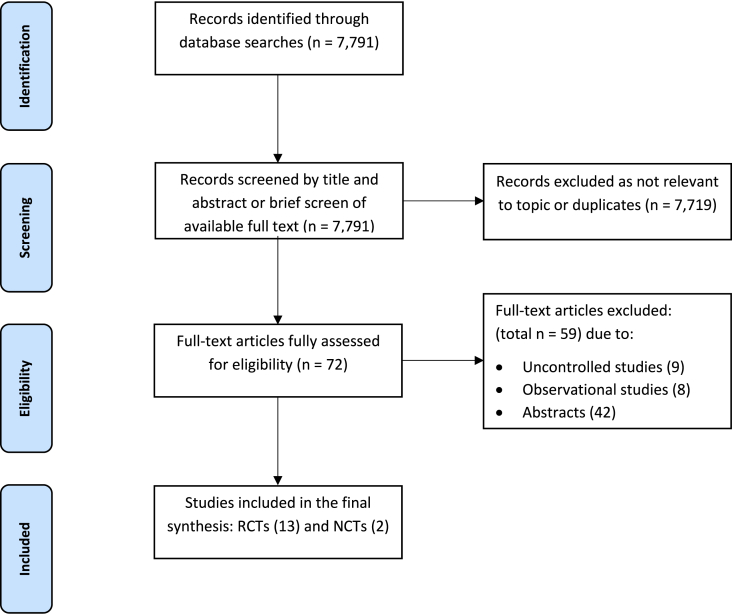
PRISMA flow diagram

Following duplicate removal, 4 reviewers (UBN, LLS, FG, MK) independently screened titles and abstracts against the eligibility criteria in pairs. Full texts of potentially eligible studies were retrieved and independently assessed by the same reviewers. Disagreements were resolved through discussion or arbitration by a third reviewer (SA). The selection process is summarised in a PRISMA flow diagram (Fig. 1).

### Data extraction

Data were independently extracted by 2 pairs of reviewers (UN, LLS, FG, MK) using a standardized form, that included: study characteristics (eg, year, country, sample size), population demographics, intervention and comparator details (eg, type, duration, dose) and outcomes (primary and secondary). Discrepancies were resolved through discussion or arbitration by a third reviewer (SA).

### Risk of bias and certainty of evidence

The risk of bias of included studies was assessed using the Cochrane Risk of Bias Tool 2 (RoB2).^34^ The certainty of evidence was evaluated using the GRADE framework^35^ and categorized as high, moderate, low, or very low. Results are presented in the Summary of findings (SoF) table (Table 1). Both assessments were carried out independently by 2 pairs of reviewers (UN, LLS, FG, MK). Disagreements were resolved through consensus or arbitration by a third reviewer (SA).

**Table 1:** Summary of findings (SOF).

Biologics in IgE-mediated food allergy: A systematic review and meta-analysis of interventional studies				
Patient or population: children and adults with confirmed food allergy Settings: primary and secondary care Intervention: biological monotherapy or combined with other types of OIT Comparison: placebo, no intervention or routine management without active treatment				
Outcomes	Relative effect (95% CI)	No of Participants (studies)	Quality of the evidence (GRADE)	Comments
**Desensitization**				
Risk ratios (RR) of desensitization following oral immunotherapy (OIT) with OMA vs control. Outcome: Tolerated 2 gr protein of 2 foods	RR 2.035 (1.29–3.22)	299 (7 studies)	⊕⊕⊕⊝ **moderate**	Downgraded for indirectness
Risk ratios (RR) of desensitization following oral immunotherapy (OIT) with OMA vs control. Outcome: Tolerated 2 gr protein of 2 foods. Sensitivity analysis	RR 1.93 (1.22–3.10)	255 (6 studies)	⊕⊕⊕⊝ **moderate**	Downgraded for indirectness
Risk ratios (RR) of desensitization following oral immunotherapy (OIT) with OMA vs control. Outcome: Increase in threshold tolerability of an allergenic food	RR 4.90 (2.14–11.20)	235 (3 studies)	⊕⊕⊕⊝ **moderate**	Downgraded for indirectness
Risk ratios (RR) of skin reactions at injection site following OMA vs placebo mono or combined therapy	RR 1.093 (0.451–2.645)	250 (4 studies)	⊕⊕⊕⊝ **moderate**	Downgraded for indirectness
Risk ratios (RR) of upper respiratory tract infection following OMA vs placebo mono or combined therapy	RR 1.272 (0.595–2.719)	98 (3 studies)	⊕⊕⊝⊝ **low**	Downgraded for indirectness and imprecision
Risk ratios (RR) of food allergy or hypersensitivity following OMA vs placebo mono or combined therapy	RR 0.554 (0.362–0.849)	299 (3 studies)	⊕⊕⊕⊝ **moderate**	Downgraded for indirectness
Risk ratios (RR) of AEs or ARs following OMA vs placebo mono or combined therapy	RR 0.913 (0.814–1.023)	271 (4 studies)	⊕⊕⊕⊝ **moderate**	Downgraded for indirectness
Risk ratios (RR) of AEs (the number of participants with AEs) following OMA vs placebo mono or combined therapy (sensitivity analysis)	RR 0.919 (0.764–1.106)	234 (3 studies)	⊕⊕⊕⊝ **moderate**	Downgraded for indirectness
Risk ratios (RR) of SAEs following OMA vs placebo mono or combined therapy	RR 0.511 (0.176–1.482)	234 (3 studies)	⊕⊕⊕⊝ **moderate**	Downgraded for indirectness

GRADE Working Group grades of evidence.

**High certainty:** Further research is very unlikely to change our confidence in the estimate of effect.

**Moderate certainty:** Further research is likely to have an important impact on our confidence in the estimate of effect and may change the estimate.

**Low certainty:** Further research is very likely to have an important impact on our confidence in the estimate of effect and is likely to change the estimate.

**Very low certainty:** We are very uncertain about the estimate.

### Data analysis and synthesis

Random-effect meta-analyses were used where possible and appropriate in Comprehensive Meta-Analysis software (version 4). Results were presented as pooled estimates with 95% confidence intervals for dichotomous outcomes.

The percentage of total variability attributable to heterogeneity between studies was quantified using the I^2^ statistic, with thresholds for interpretation according to Cochrane guidance (I^2^ < 25% indicates low, 25–50% moderate, and >50% high). The term heterogeneity used throughout the manuscript refers to this variation, not to an absolute measure of heterogeneity. Clinical and methodological heterogeneity were assessed narratively, considering factors such as study design, patient population, treatment protocols, and outcome measurements.

Subgroup and sensitivity analyses were carried out to investigate potential effect modifiers: risk of bias, comparator, type of biological therapy, treatment duration, dose variations of biologics or allergens in OIT, etc.

Funnel plots and Egger's tests for small study effects were planned but were omitted due to the limited number of eligible studies (<10). Publication bias was not formally assessed for the same reason.

## Results

### Characteristics of included studies

Our search identified 7791 potentially relevant papers, of which 7719 records were excluded as not relevant or duplicates. Furthermore, 9 uncontrolled studies, 8 observational studies, and 42 papers in an abstract format were also excluded (Supplementary materials, table E3). Thirteen RCTs satisfied our inclusion criteria and were included in the systematic review (Fig. 1). In addition, 2 relevant US National Clinical Trials (NCTs) were identified via manual search of trial repositories. In total, 15 studies^36^, ^37^, ^38^, ^39^, ^40^, ^41^, ^42^, ^43^, ^44^, ^45^, ^46^, ^47^, ^48^, ^49^, ^50^ were included: 11 RCTs,^36^, ^37^, ^38^^,^^40^^,^^41^^,^^43^, ^44^, ^45^, ^46^, ^47^, ^48^, ^49^, ^50^ 2 multiple publications,^39^^,^^42^ secondary analyses of these RCTs,^36^^,^^48^ and 2 NCTs.^49^^,^^50^ Ten of these studies were included in meta-analysis.^36^^,^^37^^,^^40^^,^^41^^,^^43^^,^^44^^,^^46^, ^47^, ^48^, ^49^

Of the 13 included studies, 9 were assessed as having low risk of bias,^36^^,^^37^^,^^40^^,^^41^^,^^43^^,^^45^^,^^47^^,^^49^^,^^50^ 3 as moderate,^38^^,^^44^^,^^48^ and 1 as high risk of bias.^46^ The studies were undertaken in Denmark (n = 1); Japan (n = 1); and the United States (n = 13). No studies carried out a cost-effectiveness analysis of biologic treatments for food allergy (Supplementary materials, table E4). A total of 1010 participants were included across the studies with an age range of 1– 60 years. Among these, 7 studies included both pediatric and adult participants,^37^^,^^40^^,^^41^^,^^44^^,^^45^^,^^48^^,^^49^ 5 studies focused exclusively on children and adolescents (0–18 years),^36^^,^^43^^,^^46^^,^^47^^,^^50^ and 1 study enrolled only adults.^38^ No differences in the treatment approach between adults and pediatric patients were observed across the studies.

### Main results

#### Desensitization

All 13 RCTs reported desensitization as an outcome. Meta-analysis was conducted on pooled data from 10 studies.^36^^,^^37^^,^^40^^,^^41^^,^^43^^,^^44^^,^^46^, ^47^, ^48^, ^49^ Three studies were excluded from meta-analysis due to heterogeneity.^38^^,^^45^^,^^50^

**Fig. 2:**
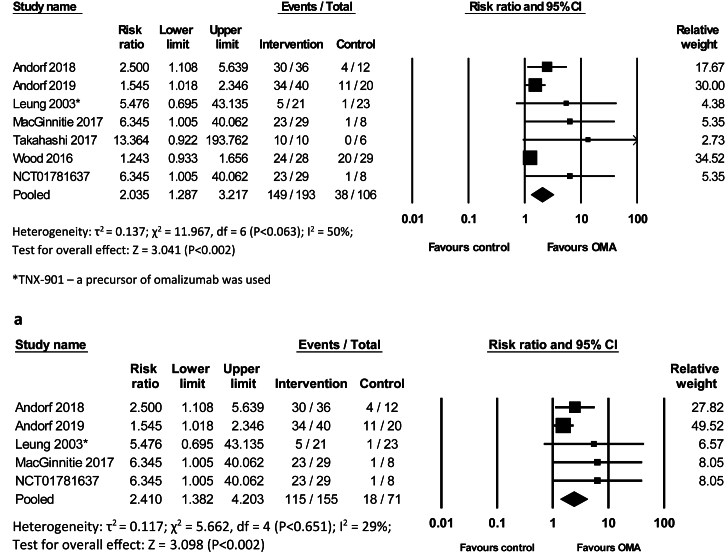
Risk Ratios (RR) of Desensitization following OMA as monotherapy or OMA with OIT vs control. Outcome: tolerated 2 gr protein of 2 foods (random-effects model). a: Risk Ratios (RR) of Desensitization OMA as monotherapy or OMA with OIT vs control. Outcome: tolerated 2 gr protein of 2 foods (random-effects model). Sensitivity analysis (LRB studies)

The meta-analysis revealed an increased likelihood of tolerating 2 g of protein from 2 foods, primarily peanut and cow's milk with OMA as monotherapy or OMA with OIT compared to control (risk ratio [RR] = 2.035, 95% CI 1.29 to 3.22, 299 participants, 7 studies, I^2^ = 50%, GRADE = moderate) (Fig. 2). Sensitivity analysis restricted to low risk of bias (LRB) supported this finding (RR = 2.41, 95% CI 1.38 to 4.2, 232 participants, 6 studies, I^2^ = 29%) (Fig. 2a). Another sensitivity analysis excluding the outlier study,^40^ as they used a precursor of OMA TNX-901, also demonstrated a similar result (RR = 1.93, 95% CI 1.22 to 3.05, 255 participants, 6 studies, I^2^ = 52%, GRADE = moderate) (Supplementary materials, figure E2b). Further analysis of only OMA with OIT vs control also demonstrated consistent findings [RR = 2.3, 95% CI 1.27 to 4.16, 182 participants, 4 studies, I^2^ = 36%] (Supplementary materials, figure E2c). Thus, meta-analyses of desensitization data showed consistently that OMA as a monotherapy or OMA with OIT vs control significantly desensitizes patients to allergenic foods.

**Fig. 3:**
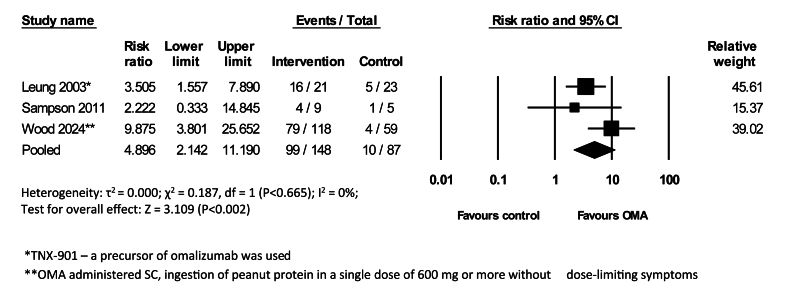
Risk Ratios (RR) of an increase in threshold tolerability of an allergenic food following OMA as a monotherapy vs control (random-effects model)

Three studies demonstrated a significant increase in threshold tolerability of allergenic food with OMA with OIT compared to control treatment (RR = 4.90, 95% CI 2.14 to 11.20, 235 participants, 3 studies, I^2^ = 0%, GRADE = moderate) (Fig. 3).

#### Sustained unresponsiveness

Only 1 study assessed sustained unresponsiveness after discontinuation of OMA and milk OIT, reporting it in 48.1% of the OMA group vs 35.7% of the placebo group at 32 months (p = 0.42).^48^

#### Adverse reactions/safety data

All included studies evaluated the safety of anti-IgE therapy with OIT in combination with, predominantly, OMA, with 1 study examining talizumab, a precursor of OMA.^40^

There was considerable heterogeneity in how safety outcomes were classified and reported. Some studies categorized adverse events (AEs) and ARs based on the organ/system affected (eg, respiratory or gastrointestinal), with or without reporting individual symptoms.^36^^,^^37^^,^^41^^,^^44^^,^^47^^,^^48^ One paper^43^ evaluating children treated with OMA as a monotherapy vs placebo reported infections such as viral infections, cystitis, and otitis media, while another paper^40^ on patients treated with TNX-901 vs placebo, categorized systemic (eg, diarrhea, nausea, fever, arthralgia) or local reactions (eg, injection-site reactions). Injection-site reactions alongside other ARs/AEs occurring during OMA with OIT were also noted in other studies.^36^^,^^37^^,^^43^^,^^47^

Nine studies addressed the severity and seriousness of AEs/ARs^36^^,^^37^^,^^40^^,^^41^^,^^43^^,^^44^^,^^46^, ^47^, ^48^ using various categorization systems. The safety profile of OMA combined with OIT was generally favorable. Most studies reported no severe or serious AEs^36^^,^^40^^,^^43^^,^^44^^,^^46^ with some reporting infections as most common AEs, with similar rates in both groups (OMA as monotherapy vs placebo).^43^ Severe reactions, when present, were reversible, did not lead to discontinuation of the study and were linked to food exposure, not OMA or placebo.^41^ Several studies noted reduced AR rates with OMA compared to placebo during OIT, particularly for reactions requiring treatment.^36^^,^^41^^,^^47^^,^^48^ Injection-site reactions were generally mild and while similar between groups (TNX-901 as monotherapy vs placebo) in 1 study,^40^ higher rates in the OMA with OIT group were reported in another.^47^

The incidence of anaphylaxis or epinephrine use was generally low, primarily reported in placebo groups.^41^^,^^48^ One study reported a higher number of doses of injectable epinephrine use for mild symptoms (throat tightness or coughing or shortness of breath) during OMA maintenance, with symptoms resolving within minutes after injection of epinephrine without further complications.^37^

#### Meta-analyses of safety data

**Fig. 4:**
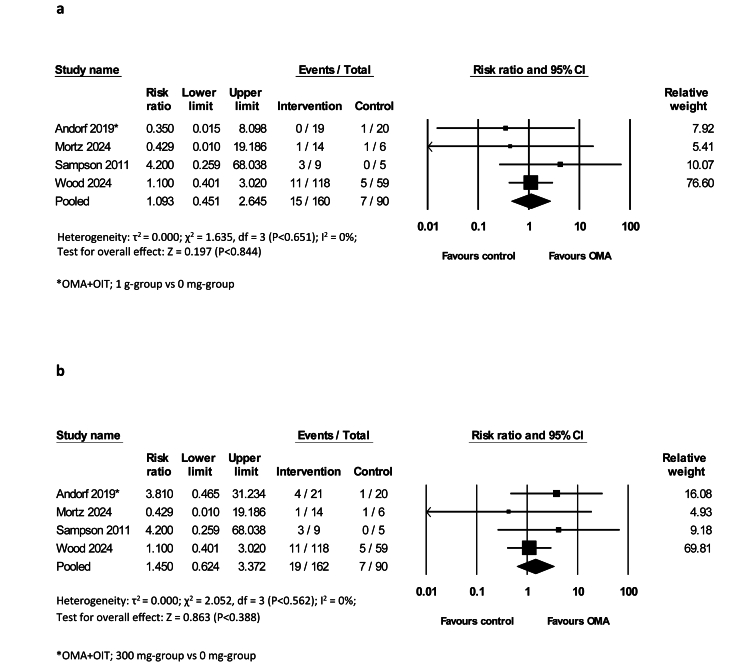
a: Risk Ratios (RR) of skin reactions at injection site following OMA as a monotherapy or OMA with OIT vs control (random-effects model) b: Risk Ratios (RR) of skin reactions at injection site following OMA as a monotherapy or OMA with OIT vs control (random-effects model)

Data from 7 RCTs^37^^,^^40^^,^^41^^,^^43^^,^^44^^,^^47^^,^^49^ were pooled to focus on OMA-attributable ARs independent of immunotherapy. No significant differences were observed in skin reactions at the injection site between the OMA and placebo with either mono or combined therapy (RR = 1.09, 95% CI 0.45 to 2.65; Fig. 4a; RR = 1.45, 95% CI 0.62 to 3.37 Fig. 4b; and SoF table). In addition, a separate analysis on the risk of skin reactions at the injection site between OMA as a monotherapy vs control demonstrated no significant differences between the 2 arms (RR = 1.13, 95% CI 0.46 to 2.76 Supplementary materials, figure E4c).

Similarly, no significant differences between OMA and placebo were observed in the analyses of AEs, such as diarrhea (RR = 3.98, 95% CI 0.74 to 21.52; RR = 1.72, 95% CI 0.24 to 12.11; RR = 1.72, 95% CI 0.24 to 12.11); nausea (RR = 1.95, 95% CI 0.65 to 5.90; RR = 1.47, 95% CI 0.46 to 4.70; RR = 1.47, 95% CI 0.46 to 4.70); vomiting (RR = 0.23, 95% CI 0.03 to 2.11; RR = 0.33, 95% CI 0.02 to 5.29 RR = 0.24, 95% CI 0.03 to 1.83); fever (RR = 1.36, 95% CI 0.19 to 9.93; RR = 2.52, 95% CI 0.44 to 14.54; RR = 1.29, 95% CI 0.18 to 9.43); upper respiratory tract infections (URTI) (RR = 1.27, 95% CI 0.60 to 2.72; RR = 1.56, 95% CI 0.78 to 3.12; RR = 0.71, 95% CI 0.27 to 1.89; RR = 1.21, 95% CI 0.55 to 2.66; RR = 1.52, 95% CI 0.74 to 3.10; RR = 0.60, 95% CI 0.21 to 1.71) across the groups receiving different doses of allergenic food, 150, 300, and 450 mg, respectively (Supplementary materials, figures E5–E9).

Risk Ratios (RR) for food allergy or hypersensitivity reactions generally favoured OMA with either mono or combined therapy over placebo across the different doses of allergenic food, 150, 300 and 450 mg and at different endpoints, 3 and 6 months respectively (RR = 0.55, 95% CI 0.36 to 0.85; RR = 0.70, 95% CI 0.36 to 1.36; RR = 0.57, 95% CI 0.36 to 0.87; RR = 0.56, 95% CI 0.37 to 0.86; RR = 0.66, 95% CI 0.32 to 1.46; RR = 0.56, 95% 0.37 to 0.86; RR = 0.52, 95% CI 0.33 to 0.82) (Supplementary materials, figures E10, a-h).

There were also no significant differences in the number of participants experiencing AEs or ARs between those receiving OMA alone or in combination with OIT and those in the control group. Furthermore, the risk ratio (RR) for AEs alone (excluding ARs) also showed no difference in safety outcomes between the intervention and control groups (Supplementary materials, figures E11a-d). Findings of analyses focusing on OMA monotherapy remained largely consistent with no or borderline differences between intervention and control groups (Supplementary materials, figures E11e-i). Serious adverse events (SAEs) were also comparable, with no statistically significant differences between OMA vs placebo in mono or combined therapy settings (Supplementary materials, figures E12a-b). Subgroup analyses for OMA monotherapy revealed similarly no differences for SAEs (Supplementary materials, figures E12c-d).

#### Immunologic outcomes

Thirteen studies assessed changes in immunological markers, including skin prick testing (SPT) reactivity, serum concentrations of specific IgE and IgG4, as well as total IgE.^36^, ^37^, ^38^^,^^40^, ^41^, ^42^, ^43^, ^44^, ^45^, ^46^, ^47^, ^48^^,^^50^ Significant reductions in SPT reactivity were consistently reported.^36^^,^^37^^,^^41^^,^^43^^,^^46^^,^^48^ Moreover, reductions in free total IgE levels indicated a reduced allergic response over the course of treatment.^40^^,^^44^ While free specific IgE was rarely measured, it is likely that the reported increases of specific IgE coincided with a decrease of free specific IgE while on OMA.^41^^,^^43^^,^^48^ Increased allergen-specific IgG4 levels relative to IgE were observed in several studies, suggesting enhanced tolerance.^36^^,^^37^^,^^43^^,^^45^^,^^46^^,^^48^ In 1 study,^45^ ratio of sIgG4:sIgE was augmented from baseline ≥25% for, at least, 2 allergens in 70% of the cases. Additional immunological benefits included reduced cytokine-producing CD4^+^ T cells (IL-4, IL-5, IL-9, IL-13), indicative of decreased inflammatory response and a reduction in ST2+ cells, involved in allergic responses.^38^ Broader immunological changes, such as increases in IgG1, IgG2, and IgA were also observed within weeks of treatment initiation,^46^ highlighting the therapeutic potential of OMA in modulating immune responses.

#### Quality of life measures

Two included studies reported QoL outcomes related to food allergy treatment using validated the FAQOL questionnaire.^45^^,^^47^ Sindher et al ^45^ reported a significant decrease in parental burden scores (p = 0.05) following OIT, with 77% of participants rating their experience with the use of biologics as “extremely positive” or “positive.” However, these results were based on a subset of participants, potentially introducing bias. Additionally, no comparative QoL outcomes were provided between the 300 mg and 1200 mg treatment groups. Conversely, Wood et al^47^ found no significant changes in QoL scores for caregivers or participants at the end of treatment compared to baseline. These contrasting findings highlight variability in QoL outcomes, potentially influenced by study design, treatment protocols, or participant characteristics.

#### Cost-effectiveness analysis

No included studies reported on the cost-effectiveness of biologics in managing food allergy. This represents a critical gap in the evidence, particularly given the high costs associated with these therapies.

### Ongoing studies

Several ongoing studies are investigating the use of biologics for treating food allergies. Ligelizumab is being evaluated in multicenter trials for peanut allergy (NCT05678959; NCT04984876), focusing on peanut protein tolerance and long-term safety and effectiveness in patients who completed Phase III trials.

Dupilumab is under investigation in a Phase 2 trial as an adjunct to OIT for cow's milk allergy, focusing on improving tolerance levels and increasing the cumulative tolerated dose of milk protein (NCT04148352). Another study investigates dupilumab as an adjunct to AR101 (peanut oral immunotherapy) in pediatric patients with peanut allergies, aiming to increase cumulative tolerated doses of peanut protein and improve safety and effectiveness outcomes (NCT03793608).

OMA remains a focal point in multiple trials across different settings. The BOOM study (Canada) evaluates its ability to accelerate time-to-maintenance in multi-food oral immunotherapy, potentially reducing the duration needed to reach maintenance doses (NCT04045301). The FASTX study (Sweden) and the OPAL study (Australia) investigate the combination of OMA and OIT for peanut allergy, focusing on improving tolerance to peanut proteins (NCT02402231; ACTRN12620001203943). Similarly, 2 other trials, including the PRROTECT study, investigate OMA as a monotherapy for peanut allergy, assessing its effectiveness in reducing peanut reactivity (NCT01781637; NCT00949078).

Additionally, the COMBINE study in the United States examines the combined use of OMA and Dupilumab to improve the success rates of passing double-blind placebo-controlled food challenges for multiple food allergies (NCT03679676).

Moreover, several smaller studies, such as those in Japan, investigate OMA's role in improving the safety and effectiveness of OIT for cow's milk allergy. These studies focus on outcomes like sustained unresponsiveness and AR rates, further contributing to understanding the broader applications of OMA in FA management (UMIN000008688; UMIN000018794, UMIN000024397) (Supplementary materials, table E2).

## Discussion

### Summary of main findings

This systematic review and meta-analysis provide moderate certainty of evidence that biologics, particularly OMA can be recommended for use in carefully selected patients with IgE-mediated food allergies as monotherapy and/or combined with OIT. We included only RCTs, as these studies represent the gold standard for evaluating effectiveness and cost-effectiveness of healthcare interventions.

We included 11 RCTs, 2 multiple reports emanating from 2 RCTs, and 2 National Clinical Trials (NCTs) encompassing 1010 participants overall. Of these, 9 RCTs were at low, 3 at moderate, and only 1 study at high risk of bias. Meta-analyses demonstrated a positive effect of OMA on achieving desensitization or increasing the tolerated threshold of the ingested food allergen compared to placebo. Importantly OMA reduced the risk of food allergic reactions compared with placebo without significant adverse or severe reactions were attributable to biologics or placebo. While the study population included both pediatric and adult participants, no significant differences in efficacy or safety outcomes were observed across age groups.

Immunological outcomes demonstrated decreased skin hypersensitivity to allergens, reduced specific IgE levels, and a significant decrease in allergic inflammation-specific cytokine-producing CD4^+^ T cells (IL-4, IL-5, Il-9, IL-13).^38^ Increased allergen-specific IgG4 levels relative to IgE were observed in several studies, suggesting enhanced tolerance.

Only 2 studies assessed QoL measures and indicated improvements among participants.^45^^,^^47^ None of studies investigated the cost-effectiveness of biologics in food allergy.

### Comparison with previous research

Our findings are broadly consistent with prior interventional, observational studies, reviews, reports, editorials, which suggest that biologics, particularly OMA appears to be a potentially promising therapeutical option for carefully selected children and adults with IgE-mediated food allergies.^18^, ^19^, ^20^, ^21^, ^22^, ^23^, ^24^, ^25^, ^26^, ^27^, ^28^, ^29^, ^30^, ^31^

Although there are limited data on OMA's use in cow's milk allergy (CMA) OIT, the World Allergy Organization (WAO) Diagnosis and Rationale for Action against Cow's Milk Allergy (DRACMA) guidelines recommend its use during the initial stages of OIT in patients with IgE-mediated CMA.^18^^,^^19^

In a rostrum, the authors concluded that FDA approval of OMA for IgE-mediated food allergies is a landmark achievement that will provide a long-awaited therapeutic option for many patients. The approval was, however, based on a relatively short-term RCT with a highly selective patient population. There is a need for robust post-approval research to evaluate^20^ its real-world effectiveness and identify patient populations most likely to benefit.

Recent observational data indicate that OMA may allow the safe reintroduction of allergenic foods in food-allergic children with severe asthma.^21^ In addition to research evidence various publications have highlighted the importance of justice in allocating OMA to patients with food allergies^23^ and have advised clinicians on its benefits and risks to optimize patient management.^22^ Yet the need for real-world studies to assess the long-term effects of biologics in food allergies and for shared decision-making in clinical consultations has been acknowledged.^25^

The ethical allocation of OMA, considering its cost and limited availability, remains a concern. While not addressed in any of the studies included in this review, economic evaluations using Markov simulation models have suggested that OMA might not be cost-effective at current prices but could become so if prices decrease or health utility gains are significant.^24^

Consistent with our findings, previous reviews concluded that while OMA provides an important option for FA, more research is needed to determine its use in clinical practice, since critical questions remain unanswered regarding the optimal duration, schedule, dosage, predictors of response and safety of OMA in long-term use.^26^, ^27^, ^28^, ^29^, ^30^, ^31^ Existing reviews also emphasize the need for standardised protocols and reporting of outcomes to facilitate evidence synthesis.^27^^,^^29^^,^^30^

### Implications for practice and research

There is emerging evidence supporting the use of biologics, particularly OMA for raising allergen tolerance thresholds in children and adults with IgE-mediated food allergies. In clinical practice, OMA can be a valuable tool for patients who fail to respond to conventional therapies.

More studies with long-term outcomes are needed to establish the effectiveness, tolerability and safety of biologics as a monotherapy or in combination with OIT. In addition, standardized definitions and reporting of AEs and ARs secondary to treatment will allow for more comprehensive analysis and comparisons. Research on cost-effectiveness and quality of life impacts is also necessary to guide patient-centred care.

### Strength and limitations

We believe that this systematic review and meta-analysis is the most robust investigation undertaken to date to support the use of biologics in IgE-mediated food allergy.^26^, ^27^, ^28^, ^29^, ^30^, ^31^ A key strength of our systematic review is the comprehensiveness of the search strategy across 10 international databases without geographical restrictions, contacting international experts in this field of research for unpublished, on-going or missing studies.

Our review differs from others by including only RCTs, which represent the highest-quality evidence, and by applying GRADE assessment to evaluate the certainty of the evidence. Overall, our findings align with previous research supporting the use of biologics, as a monotherapy or combined with OIT, for patients with IgE-mediated food allergies.

The main limitations of this systematic review stem from the heterogeneity of included populations, interventions, outcomes, diversity of biologics, OIT protocols and treatment modalities, and definition of outcomes (eg, AEs/ARs). We were also limited by the lack of data on long-term effectiveness and adverse outcomes, QoL measures and cost-effectiveness.

More research is needed to determine its use in clinical practice, since critical questions remain unanswered regarding the optimal duration, schedule, dosage, predictors of response and safety of OMA in long-term use.

## Conclusions

Based on current evidence and objective data, OMA can be recommended for carefully selected patients with IgE-mediated food allergy either as monotherapy or in combination with OIT. For cow's milk allergy its combination with OIT is particularly promising. However, patient-specific factors, such as allergen type, comorbidities, and risk of ARs, need to be addressed in order to maximise therapeutic benefits.

As ongoing, large RCTs progress, they are expected to provide critical insights to guide clinical practice and expand the indications for biologics in food allergy management. Further research should aim to optimize treatment protocols, evaluate long-term outcomes, and explore the cost-effectiveness of biologics.

## Authors’ consent for publication

Yes (from all authors).

## Availability of data and materials

The datasets generated during the current study are available from the corresponding author upon request.

## Author contributions

AF conceived this review. UN prepared the protocol, undertook searches, critical appraisal, meta-analysis and constructed summary of findings table with NTM. LLS, FG and MK undertook searches, data extractions, critical appraisal. The study was drafted by UN, NTM, LLS, FG, MK and then the manuscript was revised by all co-authors.

## Ethics statement

The study was considered exempt from ethics because it was a review of the literature and did not involve human subjects.

## Funding

World Allergy Organization provided methodology support.

## Declaration of competing interest

AF has received Speaker honoraria and advisory panel consultancy outside the submitted work for Nutricia, Abbott, Danone, Stallergenes, DBV, Novartis. Funded research (Institution) from Sanofi, Novartis, Ferrero, DBV, GSK, Astrazeneca, Hipp GmBDH, Humana SpA. IJA reports personal fees from Bayer, Bial, Cipla, Eurodrug, Faes Farma, Gebro, Glenmark, Opella, Menarini, MSD, Roxall and Sanofi outside the submitted work. SA declares that she has participated as an advisory board member, and/or consultant, and/or speaker/chair at scientific meetings for Aimmune, DBV, Ferrero, Mabylon, Novartis, Stallergenes Greer, Thermo Fisher Scientific and Ulrich outside the submitted work. Funded research (Institution) from Italian Minister of Health and Italian Minister of Education. All other authors have no conflict of interest within the scope of the submitted work.
