# Supplementary material for: Biologics in IgE-mediated food allergy: A systematic review and meta-analysis of interventional studies
Source: World Allergy Organ J. 2025 May 27;18(7):101069. doi: 10.1016/j.waojou.2025.101069 (PMC12158532; doi:10.1016/j.waojou.2025.101069)
Supplement: Multimedia component 1 [file mmc1.docx]

**Supplementary Material**

**Table E1:** **PRISMA statement for the submission for co-publication of “Biologics in Food Allergy: A systematic review and meta-analysis of interventional studies”**

| *Section/topic* | *#* | *Checklist item* | *Reported on page #* |
| --- | --- | --- | --- |
| **TITLE** | | | |
| Title | 1 | Identify the report as a systematic review, meta-analysis, or both. | 1 |
| **ABSTRACT** | | | |
| Structured summary | 2 | Provide a structured summary including, as applicable: background; objectives; data sources; study eligibility criteria, participants, and interventions; study appraisal and synthesis methods; results; limitations; conclusions and implications of key findings; systematic review registration number. | 2 |
| **INTRODUCTION** | | | |
| Rationale | 3 | Describe the rationale for the review in the context of what is already known. | 5 |
| Objectives | 4 | Provide an explicit statement of questions being addressed with reference to participants, interventions, comparisons, outcomes, and study design (PICOS). | 6 |
| **METHODS** | | | |
| Protocol and registration | 5 | Indicate if a review protocol exists, if and where it can be accessed (e.g., Web address), and, if available, provide registration information including registration number. | NA |
| Eligibility criteria | 6 | Specify study characteristics (e.g., PICOS, length of follow-up) and report characteristics (e.g., years considered, language, publication status) used as criteria for eligibility, giving rationale. | 6 |
| Information sources | 7 | Describe all information sources (e.g., databases with dates of coverage, contact with study authors to identify additional studies) in the search and date last searched. | 6 |
| Search | 8 | Present full electronic search strategy for at least one database, including any limits used, such that it could be repeated. | 6 |
| Study selection | 9 | State the process for selecting studies (i.e., screening, eligibility, included in systematic review, and, if applicable, included in the meta-analysis). | 7 |
| Data collection process | 10 | Describe method of data extraction from reports (e.g., piloted forms, independently, in duplicate) and any processes for obtaining and confirming data from investigators. | 7 |
| Data items | 11 | List and define all variables for which data were sought (e.g., PICOS, funding sources) and any assumptions and simplifications made. | 7 |
| Risk of bias in individual studies | 12 | Describe methods used for assessing risk of bias of individual studies (including specification of whether this was done at the study or outcome level), and how this information is to be used in any data synthesis. | 8 |
| Summary measures | 13 | State the principal summary measures (e.g., risk ratio, difference in means). | 8 |
| Synthesis of results | 14 | Describe the methods of handling data and combining results of studies, if done, including measures of consistency (e.g., I^2^) for each meta-analysis. | 8 |
| Risk of bias across studies | 15 | Specify any assessment of risk of bias that may affect the cumulative evidence (e.g., publication bias, selective reporting within studies). | 8 |
| Additional analyses | 16 | Describe methods of additional analyses (e.g., sensitivity or subgroup analyses, meta-regression), if done, indicating which were pre-specified. | 8 |
| *RESULTS* | | | |
| Study selection | 17 | Give numbers of studies screened, assessed for eligibility, and included in the review, with reasons for exclusions at each stage, ideally with a flow diagram. | 8 |
| Study characteristics | 18 | For each study, present characteristics for which data were extracted (e.g., study size, PICOS, follow-up period) and provide the citations. | 8 |
| Risk of bias within studies | 19 | Present data on risk of bias of each study and, if available, any outcome-level assessment (see Item 12). | 8 |
| Results of individual studies | 20 | For all outcomes considered (benefits or harms), present, for each study: (a) simple summary data for each intervention group and (b) effect estimates and confidence intervals, ideally with a forest plot. | 10-15 |
| Synthesis of results | 21 | Present results of each meta-analysis done, including confidence intervals and measures of consistency. | 10-15 |
| Risk of bias across studies | 22 | Present results of any assessment of risk of bias across studies (see Item 15). | 10-15 |
| Additional analysis | 23 | Give results of additional analyses, if done (e.g., sensitivity or subgroup analyses, meta-regression) (see Item 16). | 10-15 |
| *DISCUSSION* | | | |
| Summary of evidence | 24 | Summarize the main findings including the strength of evidence for each main outcome; consider their relevance to key groups (e.g., health care providers, users, and policy makers). | 16 |
| Limitations | 25 | Discuss limitations at study and outcome level (e.g., risk of bias), and at review level (e.g., incomplete retrieval of identified research, reporting bias). | 18 |
| Conclusions | 26 | Provide a general interpretation of the results in the context of other evidence, and implications for future research. | 19 |
| *FUNDING* | | | |
| Funding | 27 | Describe sources of funding for the systematic review and other support (e.g., supply of data); role of funders for the systematic review. | 19 |

**Search strategies**

**Search strategy 1: Medline/Embase format (***Medline, Embase, AMED. CAB, Global Health***)**

1. exp Food Hypersensitivity/
2. exp Milk Hypersensitivity/
3. exp Egg Hypersensitivity/
4. exp Peanut Hypersensitivity/
5. exp Tree nut Hypersensitivity/
6. exp Nut Hypersensitivity/
7. ((food or Oral Allergy Syndrome or milk or egg or peanut or arachis hypogaea or tree nut or hazelnut or brazil nut or walnut or chestnut or pistachio or almond or legumes or wheat or rice or soy or fish or seafood or shellfish or shrimp or lobster or crab or crawfish or kiwi or apple or peach or apricot or cherry or pear or plum or tomato or green pea or potato or carrot or parsley or celery or additives) adj3 (allerg* or hypersensitivit*)).mp.
8. Multiple allergies.mp
9. or/1-8
10. exp Desensitization, Immunologic/
11. exp Immunotherapy/
12. Desensitization.mp.
13. Hyposensitisation.mp.
14. Allergy vaccination.mp.
15. Immunotherapy.mp.
16. Oral Immunotherapy.mp.
17. Oral desensitization.mp.
18. Specific oral tolerance induction.mp.
19. Oral tolerance induction.mp.
20. Sublingual immunotherapy.mp.
21. Epicutaneous immunotherapy.mp.
22. Specific immunotherapy.mp.
23. Or/10-22
24. exp Intervention Studies/
25. Intervention Studies.mp.
26. Experimental stud*.mp.
27. exp Clinical Trial/
28. Trial.mp.
29. Clinical Trial.mp.
30. exp Controlled Clinical Trial/
31. Controlled Clinical Trial.mp.
32. Randomized Controlled Trial.mp.
33. Quasi-randomized trial.mp.
34. Non-randomized trial.mp.
35. exp Placebos/
36. Placebos.mp.
37. exp Random Allocation/
38. Random Allocation.mp.
39. exp Double-Blind Method/
40. Double-Blind Method.mp.
41. Double-Blind design.mp.
42. exp Single-Blind Method/
43. Single-Blind Method.mp.
44. Single-Blind design.mp.
45. Triple-Blind Method.mp.
46. Random*.mp.
47. Cost:.mp.
48. Cost effective:.mp.
49. Cost utility:.mp.
50. Exp Health care Costs/
51. (Costs and Costs Analysis).mp.
52. Economic evaluation*.mp.
53. ((cost effective* adj1 analys*) or cost minimization analys* or cost benefit analys* or cost utility analys* or cost consequence analys* or finances).mp.
54. Quality of life.mp.
55. Efficacy.mp.
56. Threshhold.mp.
57. Biologic*.mp.
58. (omalizumab or TNX-901 or dupilumab or reslizumab or mepolizumab or benralizumab or ligelizumab or ANB020 or etokimab or Tezepelumab or quilizumab or tralokinumab or nemolizumab).mp.
59. Or/24-58
60. 9 and 23 and 59

***Search strategy 2***: **free-field format** (*Cochrane Library, TRIP, CINAHL, ISI Web of Science, Scopus*)

(Food hypersensitivity or food allergy or Oral Allergy Syndrome or milk allergy or egg allergy or nut allergy or peanut allergy or arachis hypogaea allergy or tree nut allergy or hazelnut allergy or legumes allergy or wheat allergy or soy allergy or fish allergy or seafood allergy or shellfish allergy or kiwi allergy or apple allergy or peach allergy or additives hypersensitivity or additives allergy)

**AND**

(Immunologic, desensiti* or immunotherapy or hyposensitisation or oral immunotherapy or sublingual immunotherapy or epicutaneous immunotherapy or specific immunotherapy or oral desensiti* or Specific Oral Tolerance Induction or Oral Tolerance Induction)

**AND**

(Intervention stud* or experimental stud* or trial or clinical trial* or controlled clinical trial or randomi* controlled trial or random allocation or single blind method or double blind method or triple blind method or random* or economic evaluation* or cost effective* analys* or cost minimization analys* or cost benefit analys* or cost utility analys* or cost consequence analys* or finances or biologic* or omalizumab or TNX-901 or dupilumab or reslizumab or mepolizumab or benralizumab or ligelizumab or ANB020 or etokimab or tezepelumab or quilizumab or tralokinumab or nemolizumab)

**Table E2: List of on-going RCT studies of biologics in food allergy**

| **Author/**  **country** | **Title of study** | **Study design** | **ClinicalTrials.gov identifier** | **Population (N)/**  **sample size (n)** | **Intervention/**  **Exposure** | **Outcomes** | | **Estimated date for reporting final results** | **Notes** |
| --- | --- | --- | --- | --- | --- | --- | --- | --- | --- |
|  |  |  |  |  |  | Primary | Secondary |  |  |
| NA/Australia, Canada, France, Germany, Italy, Japan, Netherlands, Spain, United States of America | A Three-year, Multi-center, Double-blind, Extension Study to Evaluate the Long-term Safety and Efficacy of Ligelizumab in Patients Who Completed Ligelizumab's Phase III Studies in Food Allergy | UCT | NCT05678959 | 550  Age: 6-57 y | Biologic: Ligelizumab | 1. Number of treatment-emergent AEs and SAEs [Time Frame: Up to 172 weeks] | 1. Scores in the Food Allergy Independent Measure (FAIM) by age and responder [Time Frame: Day 1, 10 days before and 3 days after Week 52, 10 days before and 3 days after Week 104, 10 days before and 3 days after Week 156]  2. Scores in the Medical Outcomes Study 36-item Short Form Version 2 Acute Version (SF36v2) by age and responder [Time Frame: Day 1, 10 days before and 3 days after Week 52, 10 days before and 3 days after Week 104, 10 days before and 3 days after Week 156]  3. Number of participants tolerating a single dose of more than or equal to 600 mg of peanut protein without dose-limiting symptoms [Time Frame: Day 1, Week 52, Week 104, Week 156]  4. Scores in the Food Allergy Quality of Life Questionnaire (FAQLQ) by age and responder [Time Frame: Day 1, 10 days before and 3 days after Week 52, 10 days before and 3 days after Week 104, 10 days before and 3 days after Week 156] | February 2031 | Multicenter study. Phase 3  Sponsor: Novartis Pharmaceuticals  RECRUITING |
| NA/ Australia, Canada, Denmark, France, Germany, Italy, Japan, Netherlands, South Africa, Spain, United Kingdom, United States of America | A 52 week, multi-center, randomized, double-blind placebo-controlled study to assess the clinical efficacy and safety of ligelizumab (QGE031) in decreasing the sensitivity to peanuts in patients with peanut allergy | RCT | NCT04984876 | 211  Age 6-55 y | Biologic: Ligelizumab  Other: Placebo | 1 To evaluate the efficacy of ligelizumab 240 mg and 120 mg (SCq4w) compared to placebo, as measured by the proportion of participants who can tolerate a single dose of >= 600 mg (1044 mg cumulative tolerated dose) of peanut protein without dose-limiting symptoms during the double blind placebo controlled food challenge at Week 12 | To evaluate the efficacy of ligelizumab 240mg and 120mg (SCq4w), compared to placebo, as measured by:  -the proportion of participants who can tolerate a single dose of >= 1000mg (2044mg cum. tolerated dose) of peanut protein without dose limiting symptoms during the double blind placebo controlled food challenge (DBPCFC) at w12  - the proportion of participants who can tolerate a single dose of 3000mg (5044mg cum. tolerated dose) of peanut protein without doselimiting symptoms during the DBPCFC at w12  -the maximum symptom severity at any single challenge dose up to and including 1000mg of peanut protein during the DBPCFC at w12  -To evaluate the efficacy of 8 weeks of placebo treatment followed by 4 weeks of ligelizumab 120mg and 240mg (SCq4w) treatment compared to 12 weeks of placebo treatment, as measured by the proportion of participants who can tolerate a single dose >=1000mg of peanut protein without dose-limiting symptoms during the DBPCFC at w12 | November 2023 | Multicenter study. Phase 3  Sponsor: Novartis Pharmaceuticals  TERMINATED BUT NO RESULTS POSTED |
| Andrew J Long/ United States of America | A Phase 2, Multicenter, Randomized, Double-blind, Placebo-controlled Study to Evaluate the Efficacy and Safety of Dupilumab and Milk Oral Immunotherapy for the Treatment of Patients With Cow's Milk Allergy | RCT | NCT04148352 | 116  Age 4-50 y | Biologic: Dupilumab  Other: Placebo | Proportion of subjects treated with dupilumab plus milk protein OIT vs placebo plus milk protein OIT who tolerate at least 2040 mg (cumulative) cow's milk protein during DBPCFC to milk at week 18 [Time Frame: Week 18] | 1. Change in the cumulative tolerated dose (CTD) of milk protein during DBPCFC from baseline to week 24 across cohorts as pairwise comparisons of all treatment groups [Time Frame: Baseline and week 24]  2. Proportion of participants who tolerate at least 1040 mg cumulative milk protein at week 18 DBPCFC [Time Frame: Week 18]  3. Proportion of participants who tolerate at least 1040 mg cumulative milk protein at week 24 DBPCFC [Time Frame: week 24]  4. Proportion of participants who tolerate 4040 mg cumulative milk protein at week 24 DBPCFC [Time Frame: Week 24]  5. Change in the cumulative tolerated dose (CTD) of milk protein during DBPCFC from baseline to week 18 across cohorts as pairwise comparisons of all treatment groups [Time Frame: Baseline and week 18]  6. Proportion of participants who tolerate at least 2040 mg cumulative milk protein at week 24 DBPCFC [Time Frame: Week 24]  7. Proportion of participants who tolerate 4040 mg cumulative milk protein at week 18 DBPCFC [Time Frame: Week 18] | November 2026 | Phase 2  RECRUITING |
| Philippe Bégin, Canada | Omalizumab to Accelerate a Symptom-driven Multi-food OIT (BOOM) | RCT | NCT04045301 | 90  Age 6-25y | Biologic: Omalizumab  Other: Placebo  Other: Multi-food oral immunotherapy (OIT) | 1. To determine the efficacy of omalizumab at decreasing time-to-maintenance during a symptom-driven multi-food OIT protocol. [Time Frame: Assessed up to 52 weeks after IFE] | 1. Average up-dosing speed while on study drug. [Time Frame: From week 0 to week 12 post IFE]  2. Rate of treatment failure [Time Frame: At any time during the 12-month OIT phase]  3. Mean cumulative function of allergic adverse events attributable to food dosing throughout the trial. [Time Frame: For one year following IFE]  4. Change in reactivity threshold to food treatment mix after pre-treatment with study drug. [Time Frame: Measured 8 weeks after starting investigational product] | March 2025  RECRUITING | Phase 2  RECRUITING |
| R. Sharon Chinthrajah, United States of America | Clinical Study Using Biologics to Improve Multi OIT Outcomes (COMBINE) | RCT | NCT03679676 | 110  Age: 4-55 y | Biologic: Omaliumab  Biologic: Dupilumab  Other: placebo | 1.The success rates of passing a peanut Double-Blind Placebo Controlled Food Challenge (DBPCFC) [Time Frame: 44 weeks]  2. The success rates of passing a DBPCFC to peanut and at least one other FA [Time Frame: 44 weeks]  3. The success rates of passing a DBPCFC to peanut and two other FAs [Time Frame: 44 weeks] | 1. Proportion of participants who pass DBPCFCs for each FA at a cumulative dose of =1,043 mg, =2,043 mg, or =4,043 mg at week 32 and/or week 44. [Time Frame: week 32 and/or 44]  2. Proportion of participants who successfully pass DBPCFCs to a cumulative dose of >=1,043 mg protein to 1, 2, or 3 FAs when applicable at week 44 [Time Frame: 44 weeks]  3. Proportion of participants who have a 10-fold change in the cumulative tolerance dose for each FA at weeks 32 and/or week 44, compared to baseline [Time Frame: Baseline and week 32 and/or 44]  4. Proportion of participants who successfully pass DBPCFCs to a cumulative dose of =2,043 mg to 1, 2, or 3 FAs when applicable at week 32 [Time Frame: 32 weeks] | September 2025 | Phase 2  RECRUITING |
| Caroline Nilsson,  Sweden | Treatment of Severe Peanut Allergy With Xolair (Omalizumab) and Oral Immunotherapy (FASTX) | UCT | NCT02402231 | 23  Age 12-22 y | Biologic: Omalizumab | Peanut challenge. The peanut challenge can be positive i.e. the study object reacts to peanuts with allergic symptoms or negative i.e. no reaction in two hours after completed challenge. [Time Frame: The participants will be followed for the duration of Xolair treatment and oral immunotherapy, an expected average of 1 year] | NA | October 2020 | Unknown status |
| Shoichiro Taniuchi,Japan | Usefulness and safety of Omalizumab (anti-IgE monoclonal antibody) on oral immunotherapy for patients with severe food allergy | UCT | UMIN000008688 | 40  Age >5 y | Biologic: omalizumab | To evaluate usefulness and safety of Omalizumab(anti-IgE monoclonal antibody) on oral immunotherapy for patients with severe food allergy | NA | Unknown | Recruitment status: preinitiation |
| Robert Wood, United States of America | Omalizumab in the Treatment of Peanut Allergy | CCT | NCT00949078 | 51  Age:18- 50 y | Biologic: omalizumab | To evaluate if omalizumab is an effective treatment for peanut allergy. | To evaluate the role of allergic cells (mast cells and basophils) and IgE in food allergy. | August 2011 | Phase 2  Completed not published |
| Andrew Carr, Australia | Assessing the Utility of Combining Peanut Oral Immunotherapy and Omalizumab in Adults In Improving Tolerance of Peanut Protein with Peanut Allergy (OPAL) | UCT | ACTRN12620001203943 | 25  Age >16 y | Biologic: omalizumab | To evaluate maximum tolerated dose of peanut at Week 48 double-blind, placebo-controlled food challenge | 1. Differences in maximum tolerated peanut protein between baseline, Week 24 and Week 48 as assessed by the maximum tolerated dose of peanut protein at double-blind, placebo-controlled food challenges at these time points  2. Changes in total and specific responses in serum IgE, IgG4 and basophil reactivity in response to study interventions when compared to baseline  3. Changes in food allergy-associated quality of life parameters in participants when compared to baseline as assessed by the Food Allergy Quality of Life Questionnaire- Adult Form, and the Food Allergy Independent Measure  4. Safety of peanut oral immunotherapy as indicated by adverse reactions attributable to peanut oral immunotherapy. This will assessed by severity and number of reactions documented by the participants in their Immunotherapy Diary | Unknown | Phase 4  Approved, not yet recruiting. |
| \| Ikuya Tsuge, Japan \| \| --- \| | A pilot study to examine the effect of adjunctive anti-IgE antibody on improving the efficacy and safety of oral immunotherapy for cow's milk allergy. | UCT | UMIN000018794 | 5  Age: 6-20 y | Biologic: omalizumab | To perform oral immunotherapy (OIT) for cow's milk allergy diagnosed by oral challenge test in combination with anti-IgE therapy. | To compare frequency and severity of adverse events until reaching the target amount with conventional OIT. | Unknown | Completed, not published. Last update on March 2019 |
| Shoichiro Taniuchi, Japan | Efficacy and safety of oral immunotherapy combination with omalizumab for severe cow's milk allergy- follow up study | UCT | NCT01750879 | 15  Age: 6-15 y | Biologic: omalizumab | To evaluate the percentages of sustained unresponsiveness at the months 18 after the start of the randomized study of oral immunotherapy combination with omalizumab for severe cow's milk allergy | To evaluate the frequencies of allergic reactions from the start to the months 18 after the randomized study of oral immunotherapy combination with omalizumab for severe cow's milk allergy | Unknown | Recruitment status: preinitiation |
| Lynda Schneider, United States of America | Peanut Reactivity Reduced by Oral Tolerance in an Anti-IgE Clinical Trial (PRROTECT) | RCT | NCT01781637 | 36  Age: 7-25y | Biologic: omalizumab | Tolerance of 2000 mg 6 Weeks After Last Dose of Omalizumab/Placebo | Pass 4000 mg OFC 12 Weeks After Last Dose of Omalizumab/Placebo | May 2022 | Phase 2  Completed not published |
| NA, United States of America | Study in Pediatric Subjects With Peanut Allergy to Evaluate Efficacy and Safety of Dupilumab as Adjunct to AR101 (Peanut Oral Immunotherapy) | RCT | NCT03682770 | 148  Age 6-17y | Biologic: Dupilumab  Other: Placebo matching dupilumab  Other: AR101 | Percentage of Participants Treated With Dupilumab Plus AR101 vs Placebo Plus AR101 Who Passed a Post Up-dosing Double-blind, Placebo-controlled Food Challenge (DBPCFC) With 2044 mg (Cumulative) Peanut Protein at Visit 16 (Week 28 to 40) | 1. Change From Baseline in Cumulative Tolerated Dose (Log Transformed) of Peanut Protein During a DBPCFC at Visit 16 (Week 28 to 40) in Participants Treated With Dupilumab Plus AR101 vs Placebo Plus AR101  2. Percentage of Participants Treated With Dupilumab Plus AR101 vs Placebo Plus AR101 Who Reached the 300 mg/Day Dose of AR101 by Visit 16 (Week 28 to 40)  3. Time From Randomization to the First Time When Participants Reached the 300 mg/Day Dose of AR101 During the Up-dosing Treatment Phase by Visit 16 (Week 28 to 40)  4. Percentage of Participants (Continuously) Treated With Dupilumab Plus AR101 vs Placebo Plus AR101 Who Passed a Post Maintenance DBPCFC With 2044 mg (Cumulative) Peanut Protein at Visit 22 (Week 52 to 64)  5. Change From Baseline in Cumulative Tolerated Dose (Log Transformed) of Peanut Protein During a DBPCFC at Visit 22 (Week 52 to 64) in Participants (Continuously) Treated With Dupilumab Plus AR101 vs Placebo Plus AR101  6. Percentage of Participants (Previously) Treated With Dupilumab + AR101/Placebo +AR101 vs Placebo Plus AR101 Who Passed a Post Maintenance DBPCFC With 2044 mg (Cumulative) Peanut Protein at Visit 22 (Week 52 to 64)  7. Change From Baseline in Cumulative Tolerated Dose (Log Transformed) of Peanut Protein During a DBPCFC at Visit 22 (Week 52 to 64) in Participants (Previously) Treated With Dupilumab + AR101/Placebo +AR101 vs Placebo Plus AR101  8. Percent Change From Baseline in Peanut-specific IgE in Participants Treated With Dupilumab Plus AR101 vs Placebo Plus AR101 to Visit 16 (Week 28 to 40)  9. Percent Change From Baseline in Peanut-specific IgE in Participants (Continuously) Treated With Dupilumab Plus AR101 vs Placebo Plus AR101 to Visit 22 (Week 52 to 64) and 25 | July 2021 | Sponsor: Regeneron Pharmaceuticals  Phase 2  Completed not published |

**Table E3: Manuscripts excluded at full-text screening phase and reasons for exclusion**

| **First author** | **Year of publication** | **Reference** | **REASON FOR EXCLUSION** |
| --- | --- | --- | --- |
| Bedoret D. | 2012 | Bedoret D, Singh AK, Shaw V, et al. Changes in antigen-specific T-cell number and function during oral desensitization in cow’s milk allergy enabled with omalizumab. Mucosal Immunol. 2012;5(3):267-276. | Uncontrolled study |
| Begin P. | 2014 | Begin P, Dominguez T, Wilson SP, et al. Phase 1 results of safety and tolerability in a rush oral immunotherapy protocol to multiple foods using Omalizumab. Allergy Asthma Clin Immunol. 2014;10(1):7.doi:10.1186/1710-1492-10-7 | Uncontrolled study |
| Brandstrom J. | 2017 | Brandstrom J, Vetander M, Lilja G, et al. Individually dosed omalizumab an effective treatment for severe peanut allergy. Clin Exp Allergy. 2017;47(4):540-550 | Uncontrolled study |
| Brandstrom J. | 2019 | Brandstrom J, Vetander M, Sundqvist AC, et al. Individually dosed omalizumab facilitates peanut oral immunotherapy in peanut allergic adolescents. Clin Exp Allergy. 10AD;49(10):1328-1341. | Uncontrolled study |
| Chinuki Y. | 2023 | Chinuki Y, Kohno K, Hide M, et al. Efficacy and safety of omalizumab in adult patients with wheat-dependent exercise-induced anaphylaxis: Reduction of in vitro basophil activation and allergic reaction to wheat. Allergol. 2023;72(3):444-450. | Uncontrolled study |
| van der Heiden M. | 2021 | van der Heiden M, Nopp A, Brandstrom J, et al. A pilot study towards the immunological effects of omalizumab treatment used to facilitate oral immunotherapy in peanut-allergic adolescents. Scandinavian Journal of Immunology. 2021;93(4). e13005. | Uncontrolled study |
| Salari F. | 2022 | Salari F, Bemanian MH, Fallahpour M, et al. The Effectiveness of Oral Immunotherapy in Patients with Sesame Anaphylaxis using Omalizumab. Clin Med Res. 2022 Aug 23;20(3):125–32. | Uncontrolled study |
| Schneider LC. | 2013 | Schneider LC, Rachid R, Lebovidge J, Blood E, et al. A pilot study of omalizumab to facilitate rapid oral desensitization in high-risk peanut-allergic patients. J Allergy Clin Immunol. 2013 Dec;132(6):1368-74. | Uncontrolled study |
| Wang J. | 2023 | Wang J, Wood RA, Raymond S, Suárez-Fariñas M, Yang N, Sicherer SH, et al. Double-Blind, Placebo-Controlled Study of E-B-FAHF-2 in Combination With Omalizumab-Facilitated Multiallergen Oral Immunotherapy. J Allergy Clin Immunol Pract. 2023;11(7):2208-2216.e1. | Uncontrolled study |
| Akarcan SE | 2023 | Akarcan SE, Senol HD, Gulen F, Demir E. Food oral immunotherapy: Any distinguishing factors predicting the need of anti-IgE? Allergol Immunopathol (Madr). 2023;51(6):104-111. | Observational study |
| Andorf S, | 2017 | Andorf S, Manohar M, Dominguez T, et al. Observational long-term follow-up study of rapid food oral immunotherapy with omalizumab. Allergy Asthma Clin Immunol. 2017;13:51. | Observational study |
| Ayats-Vidal R | 2022 | Ayats-Vidal R, Riera-Rubio S, Valdesoiro-Navarrete L, et al. Long-term outcome of omalizumab-assisted desensitisation to cow’s milk and eggs in patients refractory to conventional oral immunotherapy: real-life study. Allergol Immunopathol (Madr). 2022;50(3):1-7. | Observational study |
| Azzano P | 2021 | Azzano P, Paquin M, Langlois A, et al. Determinants of omalizumab dose-related efficacy in oral immunotherapy: Evidence from a cohort of 181 patients. J Allergy Clin Immunol. 1AD;147(1):233-243 | Observational study |
| Badina L. | 2022 | Badina L, Belluzzi B, Contorno S, et al. Omalizumab effectiveness in patients with a previously failed oral immunotherapy for severe milk allergy. Immun Inflamm Dis. 1AD;10(1):117-120. | Observational study |
| Fiocchi A. | 2019 | Fiocchi A, Artesani MC, Riccardi C, Mennini M, Pecora V, Fierro V, et al. Impact of omalizumab on food allergy in patients treated for asthma: a real-life study. J Allergy Clin Immunol Pract 2019;7:1901-9. | Observational study |
| Ibanez-Sandin MD. | 2021 | Ibanez-Sandin MD, Escudero C, Candon Morillo R, et al. Oral immunotherapy in severe cow’s milk allergic patients treated with omalizumab: Real life survey from a Spanish registry. Pediatr Allergy Immunol. 8AD;32(6):1287-1295. | Observational study |
| Lee THl. | 2019 | Lee TH, Chan JKC, Lau PC, Luk WP, Fung LH. Peanut allergy and oral immunotherapy. Hong Kong Medical Journal. 2019;25(3):228-234. | Observational study |
| Yee CSK. | 2019 | Yee CSK, Albuhairi S, Noh E, El-Khoury K, Rezaei S, Abdel-Gadir A, Umetsu DT, Burke-Roberts E, lebovidge J, Schneider L, Rachid R. Long-Term Outcome of Peanut Oral Immunotherapy Facilitated Initially by Omalizumab. J Allergy Clin Immunol Pract. 2019 Feb;7(2):451-461.e7 | Observational study |
| Alba Jorda P. | 2019 | Alba Jorda P, Calaforra S, Alvarino M, Torres M, El-Qutob D. Omalizumab in spontaneous food tolerance in adult patients. Allergy: European Journal of Allergy and Clinical Immunology. 2019;74(Supplement 106):113. | Abstract |
| Arasi S. | 2017 | Arasi S, Caminiti L, Crisafulli G, Pajno GB. Omalizumab combined with oral immunotherapy for the treatment of severe cow’s milk allergy: Our 2-year-long experience. Allergy: European Journal of Allergy and Clinical Immunology. 2017;72(Supplement 103):740-741. | Abstract |
| Arasi S. | 2019 | Arasi S, Artesani MC, Riccardi C, et al. Omalizumab Gets Tolerance In Patients With Severe Food Allergy: A Real-Life Study. Journal of Allergy and Clinical Immunology. 2019;143(2 Supplement):AB271 | Abstract |
| Arasi S. | 2019 | Arasi S, Artesani MC, Riccardi C, et al. Real-life efficacy of omalizumab in children with severe allergy to multiple foods. Allergy: European Journal of Allergy and Clinical Immunology. 2019;74(Supplement 106):799. | Abstract |
| Azzano P. | 2019 | Azzano P, Paquin M, Leroux H, et al. Classification and management of gastrointestinal symptoms during omalizumab enabled oral immunotherapy. Allergy: European Journal of Allergy and Clinical Immunology. 2019;74(Supplement 106):680. | Abstract |
| Blasco-Valero C. | 2017 | Blasco-Valero C, Galvan-Blasco P, Garriga-Baraut T, Vila-Indurain B. Improvement of safety during the induction and maintenance phase of oral immunotherapy with cow’s milk and egg when assisted with omalizumab. Clinical and Translational Allergy Conference: 5th Pediatric Allergy and Asthma Meeting, PAAM. 2017;8(Supplement 2). | Abstract |
| Brandoni Petrone M. | 2023 | Brandoni Petrone M, Goyanes Malumbres M, Andrade Garban P, et al. Descriptive study on patients treated with omalizumab for severe persistent cow’s milk allergy at the hospital Universitario Fundacion Alcorcon. Allergy: European Journal of Allergy and Clinical Immunology. 2023;78(Supplement 111):469-470. | Abstract |
| Brandstrom J. | 2017 | Brandstrom J, Vetander M, Lilja G, et al. Peanut oral immunotherapy during omalizumab protection; a clinical trial on severely peanut allergic adolescents. Allergy: European Journal of Allergy and Clinical Immunology. 2017;72(Supplement 103):65-66. | Abstract |
| Demir E. | 2018 | Demir E, Eren Akarcan S, Cigerci Gunaydin N, et al. Omalizumab for patients refractory to milk oral immunotherapy. Allergy: European Journal of Allergy and Clinical Immunology. 2018;73(Supplement 105):501. | Abstract |
| Dominguez TLR. | 2013 | Dominguez TLR, Mehrotra A, Wilson S, et al. The safety of multiple oral immunotherapy in phase one studies at a single center. Clinical and Translational Allergy. 2013;3):24DUMMY. | Abstract |
| Dunham D. | 2021 | Dunham D, Zhou X, He Z, et al. Immune changes following peanut and cashew omalizumab-enabled immunotherapy in multifood allergic individuals. Allergy: European Journal of Allergy and Clinical Immunology. 2021;76(SUPPL 110):55-56. | Abstract |
| Gajare P. | 2022 | Gajare P, Cao S, Anderson B, et al. Dose-related allergic adverse events during multi-food oral immunotherapy. Journal of Allergy and Clinical Immunology. 2022;149(2 Supplement):AB34. | Abstract |
| Heiden MVD. | 2019 | Heiden MVD, Carvalho-Queiroz C, Nilsson C, Nopp A, Sverremark-Ekstrom E. The immunological signatures of success or failure following peanut oral immunotherapy combined with individualized omalizumab treatment in peanut allergic adolescents. Allergy: European Journal of Allergy and Clinical Immunology. 2019;74(Supplement 106):114. | Abstract |
| Henson M. | 2012 | Henson M, Edie A, Steele P, et al. Peanut oral immunotherapy and omalizumab treatment for peanut allergy. Journal of Allergy and Clinical Immunology. 2012;1):AB28**.** | Abstract |
| Kim JS. | 2014 | Kim JS, Wood RA, Lindblad R, et al. A randomized, double-blind, placebo-controlled trial of omalizumab combined with oral immunotherapy (OIT) in the treatment of cow’s milk allergy (CMA): Safety of dosing. Journal of Allergy and Clinical Immunology. 2014;1):AB403. | Abstract |
| Larrosa Garcia M. | 2019 | Larrosa Garcia M, Jimenez-Lozano I, Blasco-Valero C, et al. Analysis of the use of omalizumab in oral tolerance induction for high-risk food allergies in children. European Journal of Hospital Pharmacy. 2019;26(Supplement 1):A132. | Abstract |
| LeBovidge JS. | 2012 | LeBovidge JS, Haskell S, Borras I, et al. Patient and parent perspectives on quality of life during participation in a study of rapid oral desensitization with omalizumab therapy in patients with milk allergy. Journal of Allergy and Clinical Immunology. 2012;1):AB30. | Abstract |
| Le Bovidge | 2017 | Le Bovidge JS, Rachid RA, MacGinnitie A, et al. Quality of life, risk perception, and treatment burden with peanut oral immunotherapy. Journal of Allergy and Clinical Immunology. 2017;139(2 Supplement 1):AB133. | Abstract |
| Le U. | 2014 | Le U, Virkud Y, Vickery BP, et al. Omalizumab pretreatment does not protect against peanut oral immunotherapy-related adverse gastrointestinal events. Journal of Allergy and Clinical Immunology. 2014;1):AB104. | Abstract |
| Lefevre S. | 2016 | Lefevre S, Kanny G. Oral immunotherapy and omalizumab for food allergy. Allergy: European Journal of Allergy and Clinical Immunology. 2016;71(Supplement 102):269 | Abstract |
| Long AJ. | 2021 | Long AJ,Rabino G, Woch M, Schumacher C, Kost L, et al. Efficacy of eight-week omalizumab monotherapy in the desensitization of multi-food allergic patients, Abstracts from the European Academy of Allergy and Clinical Immunology Hybrid Congress, 2021,vol. 76, AB110 | Abstract |
| Mehrotra A.K. | 2012 | Mehrotra A.K., Dominguez T.L, Winterroth L, Sciancalepore A., et al. Safety of multiple allergen oral immunotherapy with and without adjunct omalizumab therapy, (2012). Abstract Book. Annals of Allergy, Asthma & Immunology, 109(5), A1–A162 | Abstract |
| Mennini, M. | 2017 | Mennini M, Artesani M.C., Dahdah L, et al. (2017). Omalizumab gives tolerance in patients with severe food allergy. Digestive and Liver Disease, 49(4), e271 | Abstract |
| Meninni M. | 2018 | Meninni M, Valluzzi RL, Fierro V, Dahdah L, et al. Omalizumab with severe food allergy: Perhaps more than a simple good idea, Journal of Pediatric Gastroenterology and Nutrition,2018, 66(Supplement 2) | Abstract |
| Nadeau KC. | 2011 | Nadeau KC, Schneider LC, Hoyte E, Borras I, Umetsu DT. Pretreatment with omalizumab permits rapid oral desensitization for cow's milk allergy, Journal of Allergy and Clinical Immunology, 127(2), AB2–AB2 | Abstract |
| Pajno GB. | 2015 | Pajno, GB; Caminiti, L; Crisafulli, G; Arasi, S; Porcaro, F, Efficacy and safety of oral immunotherapy along with omalizumab in children with severe cow’s milk allergy, (2015). Poster Discussion Session PDS 1. Allergy, 70, 113–279. | Abstract |
| Pajno GB. | 2015 | Pajno GB, Caminiti L, Crisafulli G, Arasi S, Chiera F, Salzano G; Severe Food Allergy to Cow's Milk Treated with Oral Immunotherapy Along with Omalizumab, Allergy,135:2, AB259 | Abstract |
| Peñalver Hernández MJ. | 2020 | Peñalver Hernández MJ, Moya B; Marín L, Crespo JF, Diéguez MC, Oral immunotherapy with omalizumab in patients with severe persistent allergy to cow's milk proteins, Poster session 2020, Allergy,75, AB 109 | Abstract |
| Pena Peloche M. | 2011 | Pena-Peloche M, Hinojosa-Mac ́ıas M, de la Hoz- Caballer, B, et al. Anti-IgE monoclonal antibody as a treatment of severe food allergy, Allergy Oral Abstract session, 2011,66 (Supplement s94),1-104, | Abstract |
| Pena-Peloche M. | 2011 | Peña Peloche M, Hinojosa Macías M, De La Hoz Caballer B, Terrados Cepeda S, et al. Treatment Of Severe And Persistent Food Allergy With Omalizumab. Journal of Allergy and Clinical Immunology, 2011, 127(2), AB26–AB26. | Abstract |
| Peña Peloche, M. | 2011 | Pena Peloche M, Macías MH, Cepeda ST, Gimeno PB, Sánchez Moreno GV, Álvarez-Cuesta E. Severe food allergy in children. Omalizumab as an alternative treatment to elimination diet. Clin Transl Allergy. 2011 Aug 12;1(Suppl 1):P55. | Abstract |
| Sanchez CS. | 2017 | Sanchez CS, Sanchez Chacon A, Omalizumab in desensitization food techniques, World Allergy Organization Journal 2017, 10(Suppl 1):A96 | Abstract |
| Schneider L. | 2019 | Schneider L, Rachid R, Lebovidge J, Graham D, Harrington T, Mittal M, Umetsu DT. Rapid Oral Desensitization for Severe Peanut Allergy Enabled with Omalizumab, Journal of Allergy and Clinical Immunology,131:2, AB93 | Abstract |
| Sindher S. | 2019 | Sindher S, Kumar D, Purington N, et al., Efficacy of a Fixed Dose of Omalizumab during Multi-Allergen Oral-Immunotherapy. Annals of Allergy, Asthma and Immunology. 2019; 123(5 Supplement): S50-S51. | Abstract |
| Sindher S. | 2020 | Sindher S, Long A, Purington N, Kumar D, Skura S, et al. Specific IgG4/IgE response after rapid desensitization to low-dose oral immunotherapy with adjunct omalizumab in multi-food allergic patients, World Allergy Organization Journal 2020, 13(8):OC28 | Abstract |
| Sindher S. | 2020 | Sindher S, Kumar D, Purington N, Tupa D, Long A, et al. A Phase 2 Study of Multi Oral Immunotherapy in Multi Food Allergic Patients to Test Immune Markers after Minimum Maintenance Dose using Xolair. Journal of Allergy and Clinical Immunology,2020. 145(2), AB135 | Abstract |
| Sindher S. | 2020 | Sindher S, Long A, Purington N, Kumar D, Skura S, A. Woch M, et al. Novel dosing strategy of omalizumab during multi-allergen oral-immunotherapy, World Allergy Organization Journal 2020, 13(8):OC36 | Abstract |
| Sindher S. | 2021 | Sindher S, Kumar D, Fitzpatrick J, Cao S, Long A, Woch M, Conn T, Nadeau K, MD, Chinthrajah S. A positive perception of treatment and continued adherence to dosing in a long-term follow-up study in food allergic participants undergoing multi-food oral immunotherapy. Journal of Allergy and Clinical Immunology,2021. 147:2,AB110 | Abstract |
| Sindher S. | 2022 | Sindher S, Long A, Purington N, Kumar D, Scheiber A, O’Laughlin K, et al. Alternative dosing of omalizumab as an adjunct therapy during multiallergen oral immunotherapy in food allergic patients, Journal of Allergy and Clinical Immunology,143:2, AB245 | Abstract |
| Siraj A. | 2013 | Siraj A, Sencion JOP, Sosa PP, et al., Omalizumab as adjuvant treatment in oral induction of tolerance to cow's milk. Allergy. 2013;68: 64-65 | Abstract |
| Sousa AF. | 2020 | Sousa AF, Yang A, , Mendes CG, Boufleur K, Torres P, Salles-Cunha P, Safety of Oral Immunotherapy Associated With Omalizumab In Severe Cow's Milk Allergy, J Allergy Clin Immunol 2020, 145(2),AB133 | Abstract |
| Wambre ER. | 2020 | Wambre ER, Bajzik V, Deberg H, et al., Baseline characteristics of peanut-allergic individuals during the dupilumab as adjunct to AR101 clinical trial. Allergy: European Journal of Allergy and Clinical Immunology. 2020; 75(SUPPL 109): 139 | Abstract |

**Table E4: Characteristics of included studies**

| **Study (First author, year, country)** | **Type of allergy** | **Allergy severity** | **Sample size (Male/ Female)** | **Mean age, years (SD)** | **Intervention** | **Control** | **Biological dose and duration** | **Treatment duration** | **Study duration, weeks** | **Administration route** | **Raw or cooked** | **Outcome measures** | **Risk of Bias** |
| --- | --- | --- | --- | --- | --- | --- | --- | --- | --- | --- | --- | --- | --- |
| Andorf 2018, USA | Multifood -at least two of the following: milk, egg, peanut, almond, wheat, cashew, sesame, soy, pecan, walnut, hazelnut | Moderate to severe | 48 (24/24) | OMA, 8 (7-10.3); Placebo, 7 (6-8) | OMA+OIT | Placebo | Dose per manufacturer’s instructions, S/C for 16 weeks, then discontinued | 16 W (1-8W OMA; 8-16W OMA+OIT)  16-36 W OIT | 36 W | Oral | Not specified | Primary: OFC: 2 g protein for 2 allergens.  Secondary: OFC: 4 g protein (2 foods), 2 g (3–5 foods), ADEs, time to maintenance dose, initial median tolerated dose, IgG4/IgE ratios, SPT. | Low |
| Andorf  2019, USA | Multifood -at least two of the following: milk, egg, peanut, almond, wheat, cashew, sesame, soy, pecan, walnut, hazelnut | Moderate- severe | 60 (37/23) | 1 g arm, 9.1 (2.4); 300 mg arm, 10.0 (4.0); 0 mg arm (blinded arm discontinuation), 10.3 (3.6) | OMA+OIT | Placebo (0 mg protein of each food allergen) | Dose per manufacturer’s instructions, S/C for 16 weeks, then discontinued. | Open label phase: 30 W (1-8W OMA; 8-16W OMA+OIT; OIT 16-30 W;  Randomised parallel-arm blinded phase: 6 W OIT | 36 W | Oral | Not specified | Primary: OFC: 2 g protein for 2 allergens.  Secondary: OFC: 4 g (2 allergens), 2 g (3–5 allergens), ADEs, peanut-specific IgE, IgG4, IgG4/IgE, SPT | Low |
| Chinthrajah,  2019, USA | Peanut | Not specified  Appears moderate | 20; Etokimab, 15 (8/7); Placebo  5 (2/3) | Etokimab, 27 (19–54); Placebo  22 (18–50) | Etokimab (anti IL-33) | Placebo | Single dose 300mg/100 mL i.v. | Day 1 (Etokimab administration) | 6 W | Subcutaneous (Omalizumab) | Not specified | Primary: OFC: CTD of 275 mg peanut protein.  Secondary: Proinflammatory serum cytokines (IL-4, IL-5, IL-9, IL-13), ST2, sST2, peanut/histamine-specific SPT wheal size, peanut and tIgE. | Moderate |
| Leung,  2003, USA | Peanut | Moderate- severe | 82  (45/37) | 150 mg of TNX-901, 34.9 (13-49); 300 mg of TNX-901, 28.3 (13-52); 450 mg of TNX-901, 31.6 (13-53); Placebo, 34.4 (14-59) | TNX-901 | Placebo | 150mg, 300mg, or 450mg of S/C every 4 weeks for 4 doses | 16W (4 doses of TNX-901  Every 4W) | 24 W | Subcutaneous (Omalizumab) | Raw | Primary: OFC  Secondary: Total IgE, free IgE levels, TNX-901 levels, anti-TNX-901 antibodies, ADEs. | Low |
| MacGinnitie,  2017, USA | Peanut | Not specified | 37 (22/15) | OMA, 10 (7–19); Placebo, 10 (6–17) | OMA + OIT | Placebo + OIT | Dose based on weight and baseline total IgE, S/C every 2–4 weeks. | 19W  (1–12W OMA +  12–19W OMA+OIT) | 139W | Oral | Not specified | Primary: OFC (2000 mg of proteins)  Secondary: OFC (4000 mg of proteins); sIgE levels, SPT wheal size, serious and non-serious ADEs. | Low |
| Mortz,  2024, Denmark | Multifood (cashew, hazelnut, egg, peanut, walnut) | Not specified | 20 (12/8) | OMA, 9.5 (6-16); Placebo, 13.5 (7-17) | OMA | Placebo | Dose based on weight and baseline total IgE, S/C every 2–4 weeks +/− 3 days for 3 months; non-responders received max dose (according to weight but not t-IgE) for another 3 mths | 6 months (3m initial treatment+3m for responders) | 9 months (about 39W) | Subcutaneous (Omalizumab) | Not specified | Primary: OFC  Secondary: sIgE, sIgG4;ADEs | Low |
| Sampson,  2011, USA | Peanut | Not specified | 14 (8/6) | OMA, 16.3 (11.1);  Placebo, 26.6 (22.5); | OMA | Placebo | Dose based on total IgE levels and body weight; S/C every 2 to 4 weeks for 20 to 22 weeks  Dose a minimum of 0.016 mg/kg/IgE IU/m every 4 weeks. Those requiring more than a 300mg dose had the dose divided and given every 2 weeks. | 20 to 22W OMA (m) | 24W | Subcutaneous (Omalizumab) | Raw | Primary: OFC  Secondary: sIgE, tIgE, ADEs | Moderate |
| Sindher,  2022, USA | Multifood (peanut, almond, cashew, hazelnut, walnut, salmon, cod, shrimp, egg, milk, sesame, soy, wheat) | Not specified | 60 (41/19) | OMA+mOIT (300 mg): 10 (4-20); OMA + mOIT (1200mg) 10 (4-17) | OMA + mOIT (300 mg) | OMA + mOIT (1200 mg) | 150 mg S/C every 4 wks for 3 doses | 30W (12W OMA + 18W mOIT) | 30W | Oral | Raw | Primary: Peanut sIgG4/sIgE ratio  Secondary: ADEs | Low |
| Takahashi,  2017, Japan | Cow's milk | Not specified. Appears severe | 16 (11/5) | OMA, 9.5 (8.3–10); Placebo, 9.5 (7.5–10.8) | OMA + OIT | Placebo | Dose per Genentech formula If subjects had total IgE >1500 IU/mL, they received OMA dose of 1500 IU/mL/body weight. S/C, every 2 or 4 weeks for 24 weeks | 24W  (1–8W OMA [m] + 8–24W OMA+OIT)  8W (OIT 24–32W) | 32W | Oral | Cooked | Primary: OFC  Secondary: total serum IgE levels, antigen-specific IgE, antigen-specific IgG4, SPT | High |
| Wood,  2024, USA | Multifood – at least one: peanut, cashew, egg, milk, walnut, hazelnut, wheat | Not specified | 177 (99/78) | OMA, 6.5 (4.0–11.0); Placebo, 7.0 (3.5–11.0) | OMA | Placebo | Dose and frequency based on weight and total IgE levels; S/C every 2 to 4 weeks for 16-20 weeks | 16 to 20 W | 40 to 44 W | Subcutaneous (Omalizumab) | Not specified | Primary: OFC (at least 600 mg of peanut proteins); Secondary:  OFC (at least 1000 mg for cashew, milk, and egg proteins); consumption in escalating doses up to 4000 mg of a single food, at least 2 foods, and all 3 foods; number of foods consumed at various doses (≥600 mg, ≥1000 mg, 2000 mg, or two doses of 2000 mg); quality of life, safety, SPT, basophil-activation testing. | Low |
| Wood,  2016, USA | Cow's milk | Not specified. Appears moderate | 57 (40/17) | OMA, 11.7 (9.5–15.0); Placebo 9.5 (8.0–13.2) | OMA + OIT | Placebo + OIT | Dose based on Genentech formula (0.016mg/kg/IgE IU); S/C every 2 or 4 weeks for the first 16 months of the study | 122W  (1–17W OMA + 17–122W OMA+OIT);  8W OIT (122–130W) | 139W | Oral | Cooked (Nonfat dry powdered milk) | Primary: OFC  Secondary: antigen-specific IgE, serum antigen-sIgG4, ADEs | Moderate |
| NCT01781637 | Peanut | Moderate- severe | 37 (22/15) | OMA, 10 (7-19); Placebo 10 (6-17) | OMA+ OIT | Placebo + OIT | S/C before rapid oral peanut desensitization; continued during 8 weeks, final OMA dose after peak tolerance. | NR | NR | Oral | Not specified | Primary: OFC (2000 mg of peanut proteins)  Secondary: OFC (4000 mg of peanut proteins) | Low |
| NCT03682770 | Peanut | Not specified | 148 (92/56) | Intervention,11.3 (3.12); Placebo 10.9 years (3) | Dupilumab+  AR101 | Placebo +AR101 | Participants of ≥60 kg weight (BW) – S/C 300 mg (every 2 weeks ; participants of ≥ 30kg to < 60kg - received 200 mg S/C; participants of <30kg BW received 100 mg S/C every 2 weeks. | 64W (Dupilumab 1-4W; 28-40W up dosing AR101;52-64W maintenance AR101) | 76W | Oral | Not Specified | Primary: OFC  Secondary: sIgE, sIgG4, ADEs | Low |

**T**
