# Supplementary material for: Biologics in IgE-mediated food allergy: A systematic review and meta-analysis of interventional studies
Source: World Allergy Organ J. 2025 May 27;18(7):101069. doi: 10.1016/j.waojou.2025.101069 (PMC12158532; doi:10.1016/j.waojou.2025.101069)
Supplement: Multimedia component 2 [file mmc2.pdf]

Figure E2b: Risk Ratios (RR) of Desensitisation following OIT with OMA vs control. Outcome: tolerated 2 gr protein of 2 foods (random-effects model). Sensitivity analysis

Study name	Risk ratio	Lower limit	Upper limit	Events / Total: Intervention	Events / Total: Control	Risk ratio and 95% CI	Relative weight
Andorf 2018	2.500	1.108	5.639	30 / 36	4 / 12		18.25
Andorf 2019	1.545	1.018	2.346	34 / 40	11 / 20		31.57
MacGinnitie 2017	6.345	1.005	40.062	23 / 29	1 / 8		5.42
Takahashi 2017	13.364	0.922	193.762	10 / 10	0 / 6		2.76
Wood 2016	1.243	0.933	1.656	24 / 28	20 / 29		36.58
NCT01781637	6.345	1.005	40.062	23 / 29	1 / 8		5.42
Pooled	1.927	1.218	3.050	144 / 172	37 / 83		

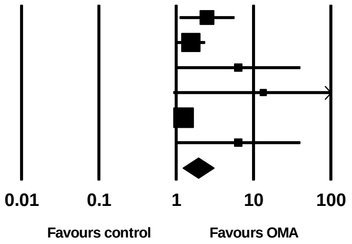

Heterogeneity: $\tau^2 = 0.129$; $\chi^2 = 10.451$, df = 5 ($P<0.063$); $I^2 = 52\%$;
Test for overall effect: Z = 2.800 ($P<0.005$)

Figure E2c: Risk Ratios (RR) of Desensitisation following OMA with OIT vs control. Outcome: tolerated 2 gr protein of 2 foods (random-effects model). Sensitivity analysis (LRB studies)

Study name	Risk ratio	Lower limit	Upper limit	Events / Total: Intervention	Events / Total: Control	Relative weight
Andorf 2018	2.500	1.108	5.639	30 / 36	4 / 12	30.13
Andorf 2019	1.545	1.018	2.346	34 / 40	11 / 20	51.89
MacGinnitie 2017	6.345	1.005	40.062	23 / 29	1 / 8	8.99
NCT01781637	6.345	1.005	40.062	23 / 29	1 / 8	8.99
Pooled	2.303	1.274	4.162	110 / 134	17 / 48	

Risk ratio and 95% CI

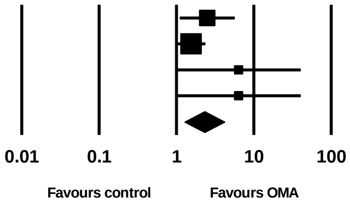

Heterogeneity: $\tau^2 = 0.130$; $\chi^2 = 4.662$, df = 3 (P<0.198); $I^2 = 36\%$;
Test for overall effect: Z = 2.762 (P<0.006)

Figure E4c: Risk Ratios (RR) of skin reactions at injection site following OMA vs control (random-effects model)

Study name	Risk ratio	Lower limit	Upper limit	Events / Total: Intervention	Events / Total: Control	Relative weight
Mortz 2024	0.429	0.032	5.779	1 / 14	1 / 6	11.75
Sampson 2011	4.200	0.259	68.038	3 / 9	0 / 5	10.26
Wood 2024	1.100	0.401	3.020	11 / 118	5 / 59	77.99
Pooled	1.130	0.463	2.756	15 / 141	6 / 70	

Risk ratio and 95% CI

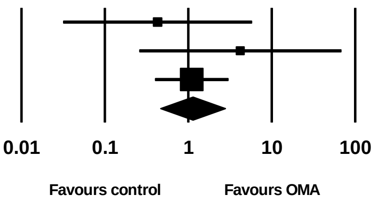

Heterogeneity: $\tau^2 = 0.000$; $\chi^2 = 1.390$, df = 2 (P<0.499); $I^2 = 0\%$;
Test for overall effect: Z = 0.268 (P<0.789)

Figure E5a: Risk Ratios (RR) of diarrhea following OMA vs control mono or combined therapy (random-effects model)

Study name	Risk ratio	Lower limit	Upper limit	Events / Total: Intervention	Events / Total: Control	Relative weight
Leung 2003*	4.842	0.590	39.755	4 / 19	1 / 23	64.28
NCT01781637	2.793	0.166	47.067	4 / 28	0 / 8	35.72
Pooled	3.978	0.736	21.517	8 / 47	1 / 31	

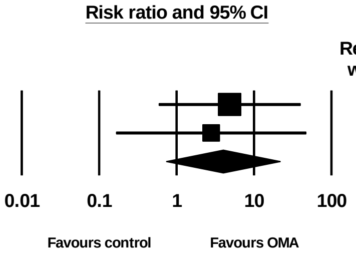

Heterogeneity: $\tau^2 = 0.000$; $\chi^2 = 0.094$, df = 1 ($P<0.760$); $I^2 = 0\%$;
Test for overall effect: Z = 1.603 ($P<0.109$)

*150 mg-group

Figure E5b: Risk Ratios (RR) of diarrhea following OMA vs control mono or combined therapy (random-effects model)

Study name	Risk ratio	Lower limit	Upper limit	Events / Total: Intervention	Events / Total: Control	Risk ratio and 95% CI	Relative weight
Leung 2003*	1.095	0.073	16.427	1 / 21	1 / 23		52.10
NCT01781637	2.793	0.166	47.067	4 / 28	0 / 8		47.90
Pooled	1.715	0.243	12.110	5 / 49	1 / 31		

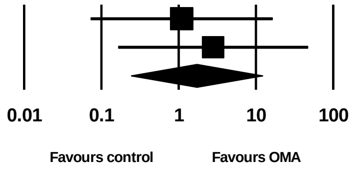

Heterogeneity: $\tau^2 = 0.000$; $\chi^2 = 0.220$, df = 1 (P<0.639); $I^2 = 0\%$;
Test for overall effect: Z = 0.541 (P<0.589)

*300 mg-group

Figure E5c: Risk Ratios (RR) of diarrhea following OMA vs control mono or combined therapy (random-effects model)

Study name	Risk ratio	Lower limit	Upper limit	Events / Total: Intervention	Events / Total: Control	Relative weight
Leung 2003*	1.095	0.073	16.427	1 / 21	1 / 23	52.10
NCT01781637	2.793	0.166	47.067	4 / 28	0 / 8	47.90
Pooled	1.715	0.243	12.110	5 / 49	1 / 31	

Risk ratio and 95% CI

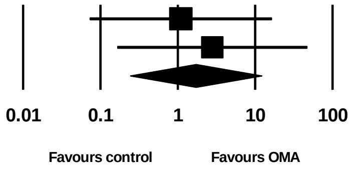

Heterogeneity: $\tau^2 = 0.000$; $\chi^2 = 0.220$, df = 1 (P<0.639); $I^2 = 0\%$;
Test for overall effect: Z = 0.541 (P<0.589)

*450 mg-group

Figure E6a: Risk Ratios (RR) of nausea following OMA vs control mono or combined therapy (random-effects model)

Study name	Risk ratio	Lower limit	Upper limit	Events / Total: Intervention	Events / Total: Control	Relative weight
Leung 2003*	3.632	0.410	32.129	3 / 19	1 / 23	25.80
NCT01781637	1.571	0.435	5.683	11 / 28	2 / 8	74.20
Pooled	1.951	0.645	5.903	14 / 47	3 / 31	

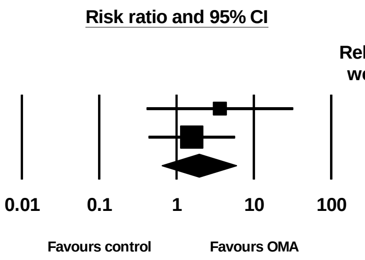

Heterogeneity: $\tau^2 = 0.000$; $\chi^2 = 0.421$, df = 1 (P<0.517); $I^2 = 0\%$;
Test for overall effect: Z = 1.183 (P<0.237)

*150 mg-group

Figure E6b: Risk Ratios (RR) of nausea following OMA vs control mono or combined therapy (random-effects model)

Study name	Risk ratio	Lower limit	Upper limit	Events / Total: Intervention	Events / Total: Control	Relative weight
Leung 2003*	1.095	0.073	16.427	1 / 21	1 / 23	18.39
NCT01781637	1.571	0.435	5.683	11 / 28	2 / 8	81.61
Pooled	1.470	0.460	4.697	12 / 49	3 / 31	

Risk ratio and 95% CI

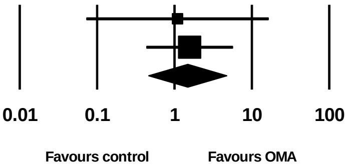

Heterogeneity: $\tau^2 = 0.000$; $\chi^2 = 0.056$, df = 1 (P<0.813); $I^2 = 0\%$;
Test for overall effect: Z = 0.651 (P<0.515)

*300 mg-group

Figure E6c: Risk Ratios (RR) of nausea following OMA vs control mono or combined therapy (random-effects model)

Study name	Risk ratio	Lower limit	Upper limit	Events / Total: Intervention	Events / Total: Control	Relative weight
Leung 2003*	1.095	0.073	16.427	1 / 21	1 / 23	18.39
NCT01781637	1.571	0.435	5.683	11 / 28	2 / 8	81.61
Pooled	1.470	0.460	4.697	12 / 49	3 / 31	

Risk ratio and 95% CI

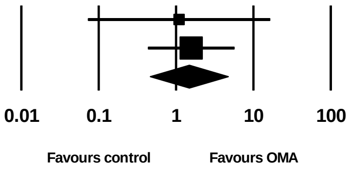

Heterogeneity: $\tau^2 = 0.000$; $\chi^2 = 0.056$, df = 1 (P<0.813); $I^2 = 0\%$;
Test for overall effect: Z = 0.651 (P<0.515)

*450 mg-group

Figure E7a: Risk Ratios (RR) of vomiting following OMA vs control mono or combined therapy (random-effects model)

Study name	Risk ratio	Lower limit	Upper limit	Events / Total: Intervention	Events / Total: Control	Relative weight
Leung 2003*	0.605	0.059	6.173	1 / 19	2 / 23	58.20
NCT01781637	0.062	0.003	1.177	0 / 28	2 / 8	41.80
Pooled	0.234	0.026	2.112	1 / 47	4 / 31	

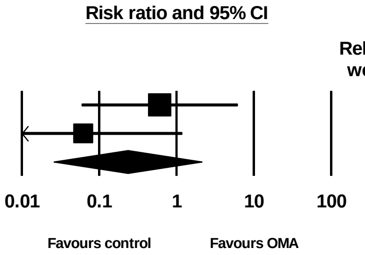

Heterogeneity: $\tau^2 = 0.076$; $\chi^2 = 1.418$, df = 1 (P<0.234); $I^2 = 29\%$;
Test for overall effect: Z = -1.294 (P<0.196)

*150 mg-group

Figure E7b: Risk Ratios (RR) of vomiting following OMA vs control mono or combined therapy (random-effects model)

Study name	Risk ratio	Lower limit	Upper limit	Events / Total: Intervention	Events / Total: Control	Relative weight
Leung 2003*	1.095	0.169	7.096	2 / 21	2 / 23	58.16
NCT01781637	0.062	0.003	1.177	0 / 28	2 / 8	41.84
Pooled	0.330	0.021	5.288	2 / 49	4 / 31	

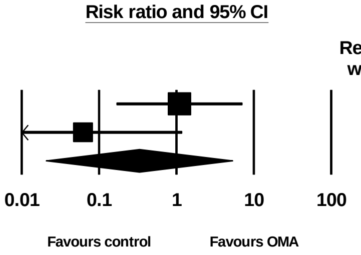

Heterogeneity: $\tau^2 = 2.538$; $\chi^2 = 2.605$, df = 1 (P<0.107); $I^2 = 62\%$;
Test for overall effect: Z = -0.784 (P<0.433)

*300 mg-group

Figure E7c: Risk Ratios (RR) of vomiting following OMA vs control mono or combined therapy (random-effects model)

Study name	Risk ratio	Lower limit	Upper limit	Events / Total: Intervention	Events / Total: Control	Relative weight
Leung 2003*	0.548	0.053	5.608	1 / 21	2 / 23	58.92
NCT01781637	0.062	0.003	1.177	0 / 28	2 / 8	41.08
Pooled	0.224	0.027	1.827	1 / 49	4 / 31	

Risk ratio and 95% CI

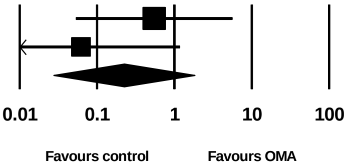

Heterogeneity: $\tau^2 = 0.539$; $\chi^2 = 1.294$, df = 1 (P<0.255); $I^2 = 23\%$;
Test for overall effect: Z = -1.397 (P<0.162)

*450 mg-group

Figure E8a: Risk Ratios (RR) of fever following OMA vs control mono or combined therapy (random-effects model)

Study name	Risk ratio	Lower limit	Upper limit	Events / Total: Intervention	Events / Total: Control	Relative weight
Leung 2003*	1.211	0.081	18.092	1 / 19	1 / 23	54.21
NCT01781637	1.552	0.082	29.435	2 / 28	0 / 8	45.79
Pooled	1.356	0.185	9.934	3 / 47	1 / 31	

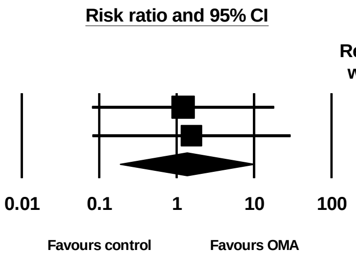

Heterogeneity: $\tau^2 = 0.000$; $\chi^2 = 0.015$, df = 1 (P<0.903); $I^2 = 0\%$;
Test for overall effect: Z = 0.300 (P<0.764)

*150 mg-group

Figure E8b: Risk Ratios (RR) of fever following OMA vs control mono or combined therapy (random-effects model)

Study name	Risk ratio	Lower limit	Upper limit	Events / Total: Intervention	Events / Total: Control	Relative weight
Leung 2003*	3.286	0.370	29.197	3 / 21	1 / 23	64.47
NCT01781637	1.552	0.082	29.435	2 / 28	0 / 8	35.53
Pooled	2.517	0.436	14.543	5 / 49	1 / 31	

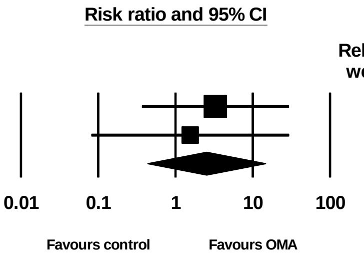

Heterogeneity: $\tau^2 = 0.000$; $\chi^2 = 1.161$, df = 1 (P<0.688); $I^2 = 0\%$;
Test for overall effect: Z = 1.031 (P<0.302)

*300 mg-group

Figure E8c: Risk Ratios (RR) of fever following OMA vs control mono or combined therapy (random-effects model)

Study name	Risk ratio	Lower limit	Upper limit	Events / Total: Intervention	Events / Total: Control	Relative weight
Leung 2003*	1.095	0.073	16.427	1 / 21	1 / 23	54.15
NCT01781637	1.552	0.082	29.435	2 / 28	0 / 8	45.85
Pooled	1.285	0.175	9.425	3 / 49	1 / 31	

Risk ratio and 95% CI

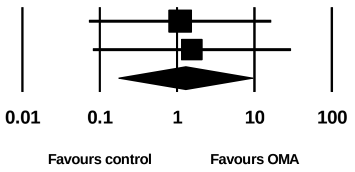

Heterogeneity: $\tau^2 = 0.000$; $\chi^2 = 1.029$, df = 1 (P<0.864); $I^2 = 0\%$;
Test for overall effect: Z = 0.247 (P<0.805)

*450 mg-group

Figure E9a: Risk Ratios (RR) of upper respiratory tract infection following OMA vs control mono or combined therapy (random-effects model)

Study name	Risk ratio	Lower limit	Upper limit	Events / Total: Intervention	Events / Total: Control	Relative weight
Leung 2003*	1.211	0.516	2.842	7 / 19	7 / 23	79.26
Mortz 2024**	1.286	0.165	9.999	3 / 14	1 / 6	13.72
NCT01781637	2.172	0.124	38.210	3 / 28	0 / 8	7.02
Pooled	1.272	0.595	2.719	13 / 61	8 / 37	

Risk ratio and 95% CI

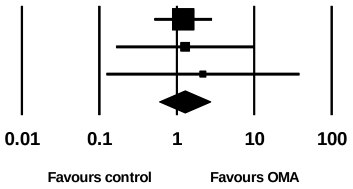

Heterogeneity: $\tau^2 = 0.000$; $\chi^2 = 0.147$, df = 2 (P<0.929); $I^2 = 0\%$;
Test for overall effect: Z = 0.620 (P<0.535)

*150 mg-group

**0-3 months

Figure E9b: Risk Ratios (RR) of upper respiratory tract infection following OMA vs control mono or combined therapy (random-effects model)

Study name	Risk ratio	Lower limit	Upper limit	Events / Total: Intervention	Events / Total: Control	Relative weight
Leung 2003*	1.565	0.729	3.357	10 / 21	7 / 23	82.68
Mortz 2024**	1.286	0.165	9.999	3 / 14	1 / 6	11.46
NCT01781637	2.172	0.124	38.210	3 / 28	0 / 8	5.86
Pooled	1.560	0.779	3.123	16 / 63	8 / 37	

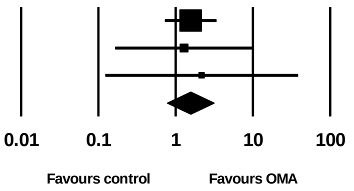

Heterogeneity: $\tau^2 = 0.000$; $\chi^2 = 0.085$, df = 2 (P<0.958); $I^2 = 0\%$;
Test for overall effect: Z = 1.255 (P<0.210)

*300 mg-group

**0-3 months

Figure E9c: Risk Ratios (RR) of upper respiratory tract infection following OMA vs control mono or combined therapy (random-effects model)

Study name	Risk ratio	Lower limit	Upper limit	Events / Total: Intervention	Events / Total: Control	Relative weight
Leung 2003*	0.469	0.139	1.584	3 / 21	7 / 23	65.29
Mortz 2024**	1.286	0.165	9.999	3 / 14	1 / 6	22.96
NCT01781637	2.172	0.124	38.210	3 / 28	0 / 8	11.75
Pooled	0.708	0.265	1.892	9 / 63	8 / 37	

Risk ratio and 95% CI

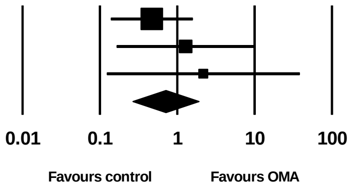

Heterogeneity: $\tau^2 = 0.000$; $\chi^2 = 1.351$, df = 2 (P<0.509); $I^2 = 0\%$;
Test for overall effect: Z = -0.688 (P<0.491)

*450 mg-group

**0-3 months

Figure E9d: Risk Ratios (RR) of upper respiratory tract infection following OMA vs control mono or combined therapy (random-effects model)

Study name	Risk ratio	Lower limit	Upper limit	Events / Total: Intervention	Events / Total: Control	Relative weight
Leung 2003*	1.211	0.516	2.842	7 / 19	7 / 23	85.29
Mortz 2024**	0.643	0.034	12.254	1 / 13	0 / 2	7.15
NCT01781637	2.172	0.124	38.210	3 / 28	0 / 8	7.56
Pooled	1.209	0.550	2.660	11 / 60	7 / 33	

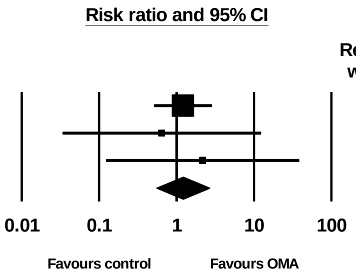

Heterogeneity: $\tau^2 = 0.000$; $\chi^2 = 0.337$, df = 2 ($P<0.845$); $I^2 = 0\%$;
Test for overall effect: $Z = 0.472$ ($P<0.637$)

*450 mg-group

**0-3 months

Figure E9e: Risk Ratios (RR) of upper respiratory tract infection following OMA vs control mono or combined therapy (random-effects model)

Study name	Risk ratio	Lower limit	Upper limit	Events / Total Intervention	Events / Total Control	Relative weight
Leung 2003*	1.565	0.729	3.357	10 / 21	7 / 23	87.87
Mortz 2024**	0.643	0.034	12.254	1 / 13	0 / 2	5.90
NCT01781637	2.172	0.124	38.210	3 / 28	0 / 8	6.23
Pooled	1.515	0.741	3.100	14 / 62	7 / 33	

Risk ratio and 95% CI

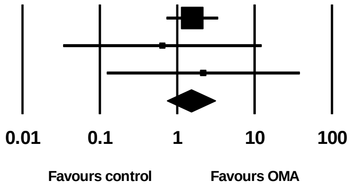

Heterogeneity: $\tau^2 = 0.000$; $\chi^2 = 0.392$, df = 2 (P<0.822); $I^2 = 0\%$;
Test for overall effect: Z = 1.138 (P<0.255)

*300 mg-group

**3-6 months

Figure E9f: Risk Ratios (RR) of upper respiratory tract infection following OMA vs control mono or combined therapy (random-effects model)

Study name	Risk ratio	Lower limit	Upper limit	Events / Total: Intervention	Events / Total: Control	Relative weight
Leung 2003*	0.469	0.139	1.584	3 / 21	7 / 23	74.06
Mortz 2024**	0.643	0.034	12.254	1 / 13	0 / 2	12.61
NCT01781637	2.172	0.124	38.210	3 / 28	0 / 8	13.33
Pooled	0.599	0.210	1.706	7 / 62	7 / 33	

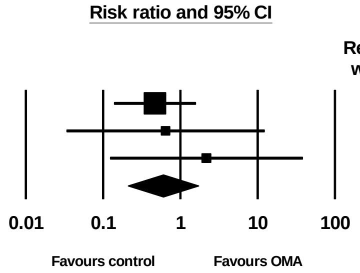

Heterogeneity: $\tau^2 = 0.000$; $\chi^2 = 0.932$, df = 2 (P<0.627); $I^2 = 0\%$;
Test for overall effect: Z = -0.960 (P<0.337)

*450 mg-group

**3-6 months

Figure E10a: Risk Ratios (RR) of food allergy or hypersensitivity following OMA vs control mono or combined therapy (random-effects model)

Study name	Risk ratio	Lower limit	Upper limit	Events / Total: Intervention	Events / Total: Control	Risk ratio and 95% CI	Relative weight
Leung 2003*	0.807	0.266	2.448	4 / 19	6 / 23		14.53
Mortz 2024**	0.429	0.032	5.779	1 / 14	1 / 6		2.64
Wood 2024	0.521	0.327	0.829	25 / 118	24 / 59		82.82
Pooled	0.552	0.362	0.843	30 / 151	31 / 88		

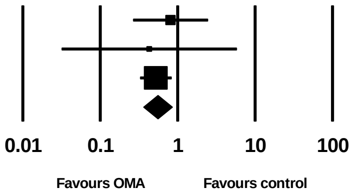

Heterogeneity: $\tau^2 = 0.000$; $\chi^2 = 0.527$, df = 2 (P<0.768); $I^2 = 0\%$;
Test for overall effect: Z = -2.715 (P<0.007)

*150 mg-group

**0-3 months

Figure E10b: Risk Ratios (RR) of food allergy or hypersensitivity following OMA vs control mono or combined therapy (random-effects model)

Study name	Risk ratio	Lower limit	Upper limit	Events / Total: Intervention	Events / Total: Control	Relative weight
Leung 2003*	1.278	0.511	3.194	7 / 21	6 / 23	32.42
Mortz 2024**	0.429	0.032	5.779	1 / 14	1 / 6	5.88
Wood 2024	0.521	0.327	0.829	25 / 118	24 / 59	61.71
Pooled	0.689	0.359	1.322	33 / 153	31 / 88	

Risk ratio and 95% CI

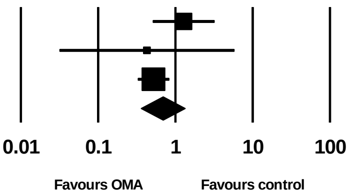

Heterogeneity: $\tau^2 = 0.126$; $\chi^2 = 2.970$, df = 2 (P<0.227); $I^2 = 33\%$;
Test for overall effect: Z = -1.051 (P<0.293)

*300 mg-group
**0-3 months

Figure E10c: Risk Ratios (RR) of food allergy or hypersensitivity following OMA as a monotherapy vs control (random-effects model)

Study name	Risk ratio	Lower limit	Upper limit	Events / Total: Intervention	Events / Total: Control	Relative weight
Leung 2003*	0.913	0.326	2.554	5 / 21	6 / 23	16.51
Mortz 2024**	0.429	0.032	5.779	1 / 14	1 / 6	2.58
Wood 2024	0.521	0.327	0.829	25 / 118	24 / 59	80.90
Pooled	0.569	0.374	0.864	31 / 153	31 / 88	

Risk ratio and 95% CI

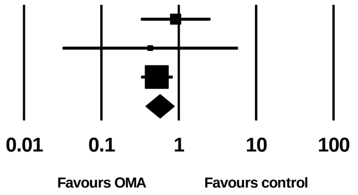

Heterogeneity: $\tau^2 = 0.000$; $\chi^2 = 0.970$, df = 2 (P<0.616); $I^2 = 0\%$;
Test for overall effect: Z = -2.611 (P<0.009)

*450 mg-group

**0-3 months

Figure E10d: Risk Ratios (RR) of food allergy or hypersensitivity following OMA as a monotherapy vs control (random-effects model)

Study name	Risk ratio	Lower limit	Upper limit	Events / Total: Intervention	Events / Total: Control	Relative weight
Leung 2003*	0.913	0.326	2.554	5 / 21	6 / 23	19.28
Mortz 2024**	0.154	0.015	1.594	1 / 13	1 / 2	3.92
Wood 2024	0.521	0.327	0.829	25 / 118	24 / 59	76.80
Pooled	0.553	0.347	0.881	31 / 152	31 / 84	

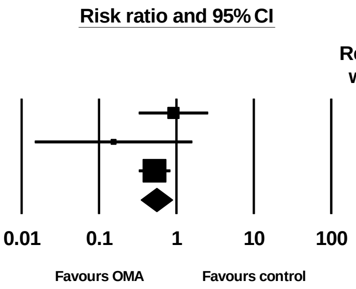

Heterogeneity: $\tau^2 = 0.000$; $\chi^2 = 1.446$, df = 2 (P<0.485); $I^2 = 0\%$;
Test for overall effect: Z = -2.678 (P<0.007)

*150 mg-group

**3-6 months

Figure E10e: Risk Ratios (RR) of food allergy or hypersensitivity following OMA as a monotherapy vs control (random-effects model)

Study name	Risk ratio	Lower limit	Upper limit	Events / Total: Intervention	Events / Total: Control	Relative weight
Leung 2003*	1.278	0.511	3.194	7 / 21	6 / 23	35.90
Mortz 2024**	0.154	0.015	1.594	1 / 13	1 / 2	10.36
Wood 2024	0.521	0.327	0.829	25 / 118	24 / 59	53.75
Pooled	0.634	0.279	1.439	33 / 152	31 / 84	

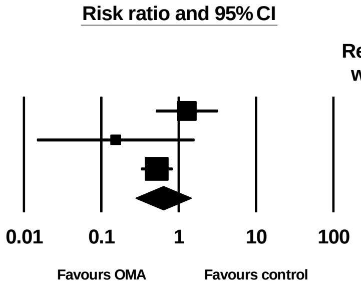

Heterogeneity: $\tau^2 = 0.193$; $\chi^2 = 3.500$, df = 2 ($P<0.174$); $I^2 = 43$;
Test for overall effect: Z = -0.979 ($P<0.328$)

*300 mg-group
**3-6 months

Figure E10f: Risk Ratios (RR) of food allergy or hypersensitivity following OMA as a monotherapy vs control (random-effects model)

Study name	Risk ratio	Lower limit	Upper limit	Events / Total: Intervention	Events / Total: Control	Relative weight
Leung 2003*	0.913	0.326	2.554	5 / 21	6 / 23	19.28
Mortz 2024**	0.154	0.015	1.594	1 / 13	1 / 2	3.92
Wood 2024	0.521	0.327	0.829	25 / 118	24 / 59	76.80
Pooled	0.553	0.347	0.881	31 / 152	31 / 84	

Risk ratio and 95% CI

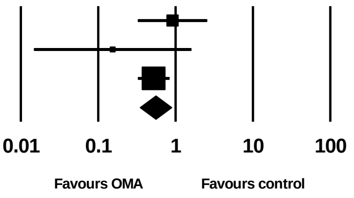

Heterogeneity: $\tau^2 = 0.000$; $\chi^2 = 1.446$, df = 2 ($P<0.485$); $I^2 = 0\%$;
Test for overall effect: Z = -2.678 ($P<0.007$)

*450 mg-group
**3-6 months

Figure E10g: Risk Ratios (RR) of food allergy or hypersensitivity following OMA as a monotherapy vs control (random-effects model) (sensitivity analysis, without Leung 2003)

Study name	Risk ratio	Lower limit	Upper limit	Events / Total: Intervention	Events / Total: Control	Relative weight
Mortz 2024*	0.429	0.032	5.779	1 / 14	1 / 6	3.09
Wood 2024	0.521	0.327	0.829	25 / 118	24 / 59	96.91
Pooled	0.518	0.328	0.818	26 / 132	25 / 65	

Risk ratio and 95% CI

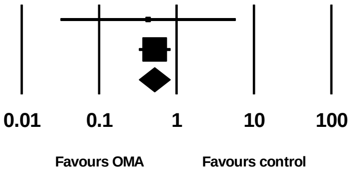

Heterogeneity: $\tau^2 = 0.000$; $\chi^2 = 0.021$, df = 1 ($P<0.885$); $I^2 = 0\%$;
Test for overall effect: $Z = -2.820$ ($P<0.005$)

*0-3 months

Figure E10h: Risk Ratios (RR) of food allergy or hypersensitivity following OMA as a monotherapy vs control (random-effects model) (sensitivity analysis, without Leung 2003)

Study name	Risk ratio	Lower limit	Upper limit	Events / Total: Intervention	Events / Total: Control	Relative weight
Mortz 2024*	0.154	0.015	1.594	1 / 13	1 / 2	4.04
Wood 2024	0.521	0.327	0.829	25 / 118	24 / 59	95.96
Pooled	0.496	0.310	0.794	26 / 131	25 / 61	

Risk ratio and 95% CI

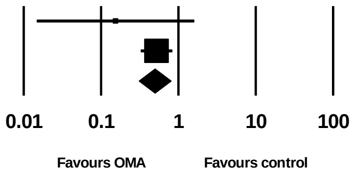

Heterogeneity: $\tau^2 = 0.004$; $\chi^2 = 1.005$, df = 1 (P<0.316); $I^2 = 0\%$;
Test for overall effect: Z = -2.921 (P<0.003)

*3-6 months

Figure E11a: Risk Ratios (RR) of AEs or ARs following OMA vs control mono or combined therapy (random-effects model)

Study name	Risk ratio	Lower limit	Upper limit	Events / Total: Intervention	Events / Total: Control	Relative weight
MacGinnitie 2017*	0.900	0.723	1.120	25 / 29	8 / 8	26.46
Mortz 2024**	1.500	0.431	5.220	7 / 14	2 / 6	0.84
Wood 2024	0.815	0.672	0.988	75 / 118	46 / 59	33.73
NCT01781637	1.006	0.841	1.202	28 / 29	8 / 8	38.98
Pooled	0.913	0.814	1.023	135 / 190	64 / 81	

Risk ratio and 95% CI

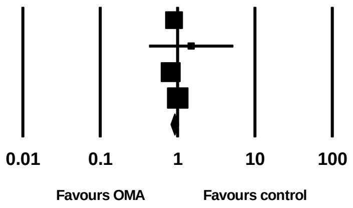

Heterogeneity: $\tau^2 = 0.000$; $\chi^2 = 3.088$, df = 3 ($P<0.378$); $I^2 = 3\%$;
Test for overall effect: $Z = -1.562$ ($P<0.118$)

*Total reactions for entire study
**0-3 months

Figure E11b: Risk Ratios (RR) of AEs or ARs following OMA vs control mono or combined therapy (random-effects model)

Study name	Risk ratio	Lower limit	Upper limit	Events / Total: Intervention	Events / Total: Control	Relative weight
MacGinnitie 2017*	0.900	0.723	1.120	25 / 29	8 / 8	26.46
Mortz 2024**	1.500	0.431	5.220	7 / 14	2 / 6	0.84
Wood 2024	0.815	0.672	0.988	75 / 118	46 / 59	33.73
NCT01781637	1.006	0.841	1.202	28 / 29	8 / 8	38.98
Pooled	0.913	0.814	1.023	135 / 190	64 / 81	

Risk ratio and 95% CI

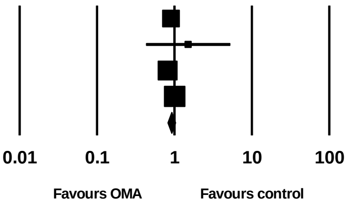

Heterogeneity: $\tau^2 = 0.000$; $\chi^2 = 2.599$, df = 3 (P<0.458); $I^2 = 0\%$;
Test for overall effect: Z = -1.689 (P<0.091)

*Total reactions for entire study
**3-6 months

Figure E11c: Risk Ratios (RR) of AEs (the number of participants with AEs) following OMA vs control mono or combined therapy (sensitivity analysis) (random-effects model)

Study name	Risk ratio	Lower limit	Upper limit	Events / Total: Intervention	Events / Total: Control	Relative weight
Mortz 2024*	1.500	0.431	5.220	7 / 14	2 / 6	2.15
Wood 2024	0.815	0.672	0.988	75 / 118	46 / 59	47.10
NCT01781637	1.006	0.841	1.202	28 / 29	8 / 8	50.75
Pooled	0.919	0.764	1.106	110 / 161	56 / 73	

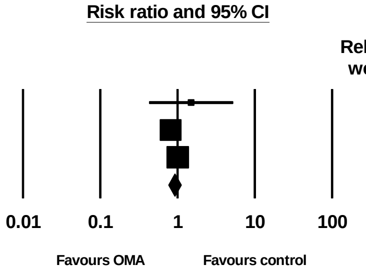

Heterogeneity: $\tau^2 = 0.009$; $\chi^2 = 3.065$, df = 2 (P<0.216); $I^2 = 35\%$;
Test for overall effect: Z = -0.896 (P<0.370)

*0-3 months

Figure E11d: Risk Ratios (RR) of AEs (the number of participants with AEs) following OMA vs control mono or combined therapy (sensitivity analysis) (random-effects model)

Study name	Risk ratio	Lower limit	Upper limit	Events / Total: Intervention	Events / Total: Control	Relative weight
Mortz 2024*	1.500	0.102	22.054	3 / 13	0 / 2	0.38
Wood 2024	0.815	0.672	0.988	75 / 118	46 / 59	47.44
NCT01781637	1.006	0.841	1.202	28 / 29	8 / 8	52.19
Pooled	0.912	0.773	1.075	106 / 160	54 / 69	

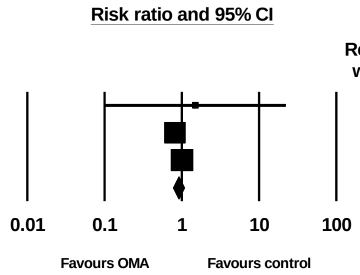

Heterogeneity: $\tau^2 = 0.005$; $\chi^2 = 2.593$, df = 2 (P<0.273); $I^2 = 23\%$;
Test for overall effect: Z = -1.097 (P<0.273)

*3-6 months

Figure E11e: Risk Ratios (RR) of AEs or ARs following OMA vs control (random-effects model)

Study name	Risk ratio	Lower limit	Upper limit	Events / Total: Intervention	Events / Total: Control	Relative weight
Mortz 2024*	1.500	0.431	5.220	7 / 14	2 / 6	2.33
Wood 2024	0.815	0.672	0.988	75 / 118	46 / 59	97.67
Pooled	0.827	0.684	1.000	82 / 132	48 / 65	

Risk ratio and 95% CI

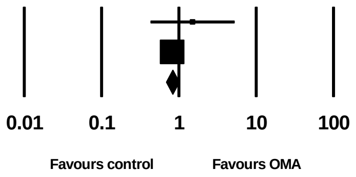

Heterogeneity: $\tau^2 = 0.000$; $\chi^2 = 0.897$, df = 1 ($P<0.344$); $I^2 = 0\%$;
Test for overall effect: Z = -1.958 ($P<0.050$)

*0-3 months

Figure E11f: Risk Ratios (RR) of AEs or ARs following OMA vs control (random-effects model)

Study name	Risk ratio	Lower limit	Upper limit	Events / Total: Intervention	Events / Total: Control	Relative weight
Mortz 2024*	0.692	0.154	3.119	3 / 13	2 / 6	1.61
Wood 2024	0.815	0.672	0.988	75 / 118	46 / 59	98.39
Pooled	0.813	0.672	0.984	78 / 131	48 / 65	

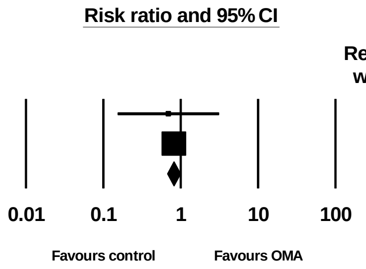

Heterogeneity: $\tau^2 = 0.000$; $\chi^2 = 0.045$, df = 1 (P<0.833); $I^2 = 0\%$;
Test for overall effect: Z = -2.124 (P<0.034)

*3-6 months

Figure E11g: Risk Ratios (RR) of AEs or ARs following OMA with OIT vs control (random-effects model)

Study name	Risk ratio	Lower limit	Upper limit	Events / Total: Intervention	Events / Total: Control	Relative weight
MacGinnitie 2017*	0.900	0.723	1.120	25 / 29	8 / 8	40.03
Wood 2024	1.006	0.841	1.202	28 / 29	8 / 8	59.97
Pooled	0.962	0.838	1.105	53 / 58	16 / 16	

Risk ratio and 95% CI

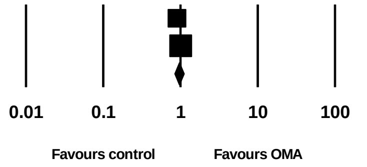

Heterogeneity: $\tau^2 = 0.000$; $\chi^2 = 0.597$, df = 1 (P<0.440); $I^2 = 0\%$;
Test for overall effect: Z = -0.548 (P<0.583)

*Total reactions for entire study

Figure E11h: Risk Ratios (RR) of AEs following OMA vs control (random-effects model)

Study name	Risk ratio	Lower limit	Upper limit	Events / Total: Intervention	Events / Total: Control	Relative weight
Mortz 2024*	1.500	0.431	5.220	7 / 14	2 / 6	2.33
Wood 2024	0.815	0.672	0.988	75 / 118	46 / 59	97.67
Pooled	0.827	0.684	1.000	82 / 132	48 / 65	

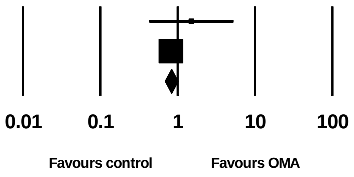

Heterogeneity: $\tau^2 = 0.000$; $\chi^2 = 0.897$, df = 1 (P<0.344); $I^2 = 0\%$;
Test for overall effect: Z = -1.958 (P<0.050)

*0-3 months

Figure E11i: Risk Ratios (RR) of AEs following OMA vs control (random-effects model)

Study name	Risk ratio	Lower limit	Upper limit	Events / Total: Intervention	Events / Total: Control	Relative weight
Mortz 2024*	1.500	0.102	22.054	3 / 13	0 / 2	0.51
Wood 2024	0.815	0.672	0.988	75 / 118	46 / 59	99.49
Pooled	0.818	0.675	0.991	78 / 131	46 / 61	

Risk ratio and 95% CI

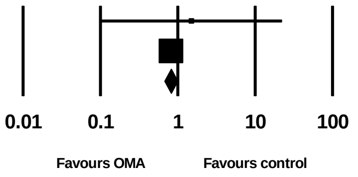

Heterogeneity: $\tau^2 = 0.000$; $\chi^2 = 0.197$, df = 1 (P<0.657); $I^2 = 0\%$;
Test for overall effect: Z = -2.053 (P<0.040)

*3-6 months

Figure E12a: Risk Ratios (RR) of SAEs following OMA vs control mono or combined therapy (random-effects model)

Study name	Risk ratio	Lower limit	Upper limit	Events / Total: Intervention	Events / Total: Control	Relative weight
Mortz 2024*	0.429	0.032	5.779	1 / 14	1 / 6	15.38
Wood 2024	1.500	0.159	14.111	3 / 118	1 / 59	20.71
NCT01781637	0.368	0.103	1.318	4 / 29	3 / 8	63.91
Pooled	0.504	0.182	1.397	8 / 161	5 / 73	

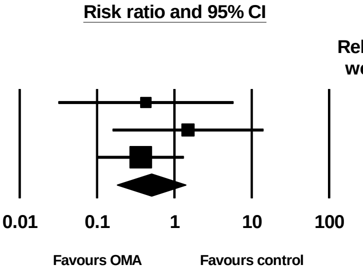

Heterogeneity: $\tau^2 = 0.000$; $\chi^2 = 1.150$, df = 2 (P<0.563); $I^2 = 0\%$;
Test for overall effect: Z = -1.236 (P<0.217)

*0-3 months

Figure E12b: Risk Ratios (RR) of SAEs following OMA vs control mono or combined therapy (random-effects model)

Study name	Risk ratio	Lower limit	Upper limit	Events / Total: Intervention	Events / Total: Control	Relative weight
Mortz 2024*	0.154	0.015	1.594	1 / 13	1 / 2	18.36
Wood 2024	1.500	0.159	14.111	3 / 118	1 / 59	19.98
NCT01781637	0.368	0.103	1.318	4 / 29	3 / 8	61.66
Pooled	0.415	0.152	1.130	8 / 160	5 / 69	

Risk ratio and 95% CI

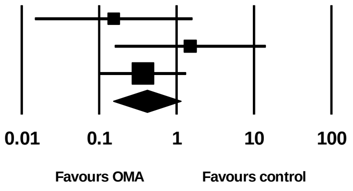

Heterogeneity: $\tau^2 = 0.000$; $\chi^2 = 1.536$, df = 2 (P<0.464); $I^2 = 0\%$;
Test for overall effect: Z = -1.405 (P<0.160)

*3-6 months

Figure E12c: Risk Ratios (RR) of SAEs following OMA vs control (random-effects model)

Study name	Risk ratio	Lower limit	Upper limit	Events / Total: Intervention	Events / Total: Control	Relative weight
Mortz 2024*	0.429	0.032	5.779	1 / 14	1 / 6	42.61
Wood 2024	1.500	0.159	14.111	3 / 118	1 / 59	57.39
Pooled	0.880	0.161	4.806	4 / 132	2 / 65	

Risk ratio and 95% CI

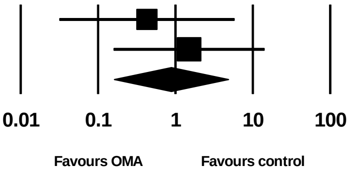

Heterogeneity: $\tau^2 = 0.000$; $\chi^2 = 0.511$, df = 1 (P<0.475); $I^2 = 0\%$;
Test for overall effect: Z = -0.148 (P<0.882)

*0-3 months

Figure E12d: Risk Ratios (RR) of SAEs following OMA vs control (random-effects model)

Study name	Risk ratio	Lower limit	Upper limit	Events / Total: Intervention	Events / Total: Control	Relative weight
Mortz 2024*	0.154	0.015	1.594	1 / 13	1 / 2	48.89
Wood 2024	1.500	0.159	14.111	3 / 118	1 / 59	51.11
Pooled	0.493	0.053	4.587	4 / 131	2 / 61	

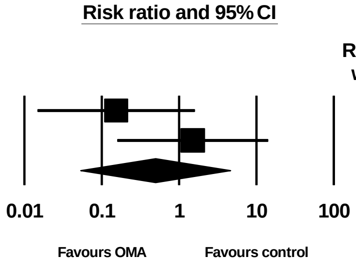

Heterogeneity: $\tau^2 = 1.227$; $\chi^2 = 1.899$, df = 1 (P<0.475); $I^2 = 47\%$;
Test for overall effect: Z = -0.622 (P<0.534)

*3-6 months
